# A Practical Evaluation of a High-Security Energy-Efficient Gateway for IoT Fog Computing Applications

**DOI:** 10.3390/s17091978

**Published:** 2017-08-29

**Authors:** Manuel Suárez-Albela, Tiago M. Fernández-Caramés, Paula Fraga-Lamas, Luis Castedo

**Affiliations:** Department Computer Engineering, Faculty of Computer Science, Universidade da Coruña, 15071 A Coruña, Spain; tiago.fernandez@udc.es (T.M.F.-C.); paula.fraga@udc.es (P.F.-L.); luis.castedo@udc.es (L.C.)

**Keywords:** ECC, ECDSA, RSA, ECDHE, IoT, IoT gateway, TLS, power consumption, performance, IoT security, cryptographic security, energy efficiency, fog computing

## Abstract

Fog computing extends cloud computing to the edge of a network enabling new Internet of Things (IoT) applications and services, which may involve critical data that require privacy and security. In an IoT fog computing system, three elements can be distinguished: IoT nodes that collect data, the cloud, and interconnected IoT gateways that exchange messages with the IoT nodes and with the cloud. This article focuses on securing IoT gateways, which are assumed to be constrained in terms of computational resources, but that are able to offload some processing from the cloud and to reduce the latency in the responses to the IoT nodes. However, it is usually taken for granted that IoT gateways have direct access to the electrical grid, which is not always the case: in mission-critical applications like natural disaster relief or environmental monitoring, it is common to deploy IoT nodes and gateways in large areas where electricity comes from solar or wind energy that charge the batteries that power every device. In this article, how to secure IoT gateway communications while minimizing power consumption is analyzed. The throughput and power consumption of Rivest–Shamir–Adleman (RSA) and Elliptic Curve Cryptography (ECC) are considered, since they are really popular, but have not been thoroughly analyzed when applied to IoT scenarios. Moreover, the most widespread Transport Layer Security (TLS) cipher suites use RSA as the main public key-exchange algorithm, but the key sizes needed are not practical for most IoT devices and cannot be scaled to high security levels. In contrast, ECC represents a much lighter and scalable alternative. Thus, RSA and ECC are compared for equivalent security levels, and power consumption and data throughput are measured using a testbed of IoT gateways. The measurements obtained indicate that, in the specific fog computing scenario proposed, ECC is clearly a much better alternative than RSA, obtaining energy consumption reductions of up to 50% and a data throughput that doubles RSA in most scenarios. These conclusions are then corroborated by a frame temporal analysis of Ethernet packets. In addition, current data compression algorithms are evaluated, concluding that, when dealing with the small payloads related to IoT applications, they do not pay off in terms of real data throughput and power consumption.

## 1. Introduction

The Internet of Things (IoT) refers to a paradigm where physical devices (e.g., home appliances, environmental sensors and actuators, vehicles) are interconnected using a communication network that allows for real-time data exchange and control. Smart environments rely on the constant availability of sensor and actuator devices, whose power consumption is a concern due to the large number of sensor nodes to be deployed. IoT networks make possible the required integration and connectivity with already available networks, thus enabling sensor networks to reach integration levels impossible to achieve with traditional approaches.

Cloud computing has been a success thanks to offloading clients from computational-intensive tasks. However, in scenarios where latency and communications have to be minimized, other paradigms have arisen by moving the capabilities of the cloud towards the edge of the network [1]. Fog computing is one of such paradigms, being regarded as an extension of cloud computing where part of the computational and communication capabilities of the cloud are moved close to the sensor nodes [2]. Such a movement derives on the following remarkable benefits [3]:

- Latency minimization allows for providing new IoT real-time applications.
- The fog distributes computational and storing resources, which is ideal for large and widely distributed sensor networks.
- The resource distribution also improves mobility and location awareness, providing services to mobile and location constrained users.
- The fog connects devices in different physical environments, enabling their interaction, which may lead to provide new services and functionality.
- The fog is highly flexible, being really easy to scale the network.

To provide such benefits, an IoT fog computing system requires three elements: IoT nodes, IoT gateways and a cloud. IoT nodes are usually composed of one or more sensors (e.g., temperature, relative humidity, human presence, CO${}_{2}$ level), a wired or wireless transceiver, a computing device (e.g., a microcontroller, an ASIC (Application-Specific Integrated Circuit), a SoC (System-on-Chip)) and a power source. The cloud is basically a server or a set of servers with large computational power and storing capabilities that receives, processes and analyzes all the data collected from the IoT nodes. IoT gateways connect the IoT nodes with the cloud and among them.

In terms of energy efficiency, researchers have traditionally focused on the nodes, since they are usually battery operated [4], but, in the last few years, the energy consumption on the cloud has been also thoroughly studied [5,6]. There is also some research on the energy efficiency of the IoT gateways [7], but it is usually assumed that they have direct access to the grid, which is not always the case: in applications for precision agriculture [8], smart cities [9], and Industry 4.0 [10,11], it is common to deploy IoT nodes and gateways in large areas where a power outlet is not available and electricity has to be harvested to charge the batteries that power every device. In addition, note that the main target of fog computing is the reduction of both the computing and networking energy consumptions through adaptive horizontal (e.g., intra-fog nodes) and vertical (e.g., inter-fog nodes) scaling of the overall available resource pool [12].

IoT gateways can be improved in different ways to reduce power consumption, but, in this paper, how to increase energy efficiency and maximize data throughput while guaranteeing high security in the communications between IoT gateways and with the cloud is analyzed. Note that, although this improvement in the energy efficiency is especially useful for battery-operated gateways, the increase on the level of security may also benefit other IoT applications like home automation [13,14,15], defense and public safety [16], transportation and connected vehicles [17], or healthcare, where security issues can pose risks for human safety and privacy [18,19] and which can be the target of cyberwarfare attacks [20].

It is also important to emphasize that security in IoT and fog computing systems is often overlooked or not completely addressed [18,21,22], slowing down the broad adoption of IoT. Furthermore, one of the main barriers for not implementing security mechanisms to protect the communications is the low computing capabilities of most IoT nodes, which is a topic to be researched in the next years.

In comparison to the cloud or to regular computers, IoT gateways and nodes are resource-constrained and usually embed low-cost computing devices that consume little power [23]. Resource-constrained devices are designed limiting their memory, storing capacity and processing power, as well as their network communication capabilities, including their data rate, in order to reduce power consumption. In the case of a Wireless Sensor Networks (WSN), low-power consumption requirements make difficult the implementation of the complex and heavy operations required by ciphering algorithms to encrypt and secure communications [24,25,26,27]. Thus, securing IoT communications at IoT gateways must be addressed carefully to achieve a good trade-off between security, energy efficiency and performance.

Since IoT fog computing networks usually rely on Internet protocols, the use of already existing and proven security protocols seems to be the best approach in terms of reliability and implementation efficiency. As explained in [16], the fog layer must provide horizontal integration between different layer protocols. IoT nodes and intermediate devices implement heterogeneous protocols; thus, the IoT fog paradigm relies on the gateways to translate between them and allow for the data aggregation needed to provide the required services. For this reason, IoT fog gateways are assumed to support Transmission Control Protocol/Internet Protocol (TCP/IP).

**Figure 1:**
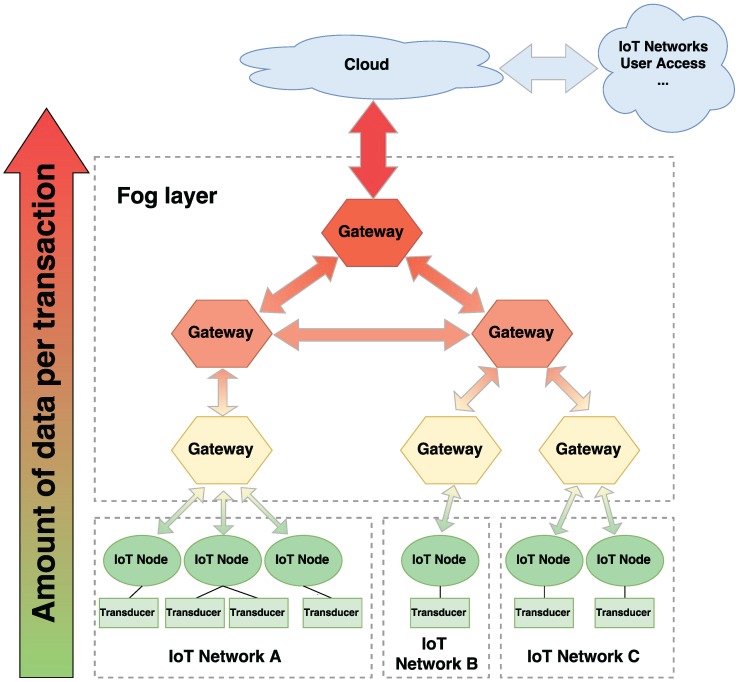
Generic Internet of Things (IoT) fog computing architecture.

A generic IoT fog computing architecture considering all of these aspects is described in Figure 1. The scenario presented considers three different IoT networks and a fog layer that allows for the communications among the nodes between the networks and to the cloud. The bidirectional arrows between the different elements represent data transmissions. Three layers of gateways were considered, the one placed at the top being responsible for providing a single access point to the fog. This layered approximation works similarly to the different cache memory levels in a computer, which are designed to reduce the latency for accessing the processor memory. In this case, the layer closest to the IoT nodes is the one that responds faster, but it has less computational and memory resources. Services are then distributed among the gateways in the fog layer. Depending on each service need (e.g., latency, computational capacity, data aggregation), they will be deployed closer to the nodes or to the cloud.

It is important to note that, since IoT networks adopt heterogeneous topologies, the architecture presented in Figure 1 does not fit in every possible IoT deployment. Nevertheless, the vast majority of IoT fog computing systems will follow this architecture to some extent. From bottom-up, the amount of data transmitted with each transaction grows, as well as the computational capabilities of the gateways. This increase in data throughput and computational capabilities as we get closer to the top layers is represented by the big arrow on the left. The requests exchanged between IoT nodes from the same network consist of small payloads, whereas transactions between different IoT networks are usually larger, since protocol translations may be needed. Furthermore, IoT gateways can aggregate information collected from several IoT nodes, allowing the top layers to provide more complex services. Therefore, the first layer of the fog transmits more data than the IoT nodes, but less than the second upper layer of gateways. This leads the IoT fog layer to exchange payloads with a wide range of sizes.

When securing IoT devices and its communications, several new challenges have to be addressed since the topology and size of the networks, as well as the communication schemes, are new and not completely explored. IoT deployments have some unique characteristics that have a direct impact on the security of the data and the communications involved:

- Resource-constrained end- and intermediate-devices: the devices forming IoT networks are constrained in terms of available data storage and computational capabilities. Security mechanisms must be aware of these limitations, reducing both the need for data storage and the computational load of the required algorithms.
- Number of devices: IoT networks are formed by thousands of devices with heterogeneous communication needs. The transmission of data between devices tends to be asynchronous, creating high requirements for backend and middleware applications in terms of availability and data throughput. Gateways and backend servers have to be capable of handling very large numbers of secure connections, and at the same time processing the relevant data to provide the necessary services.
- Scalability: IoT networks tend to grow very fast. The infrastructure supporting them has to be able to cope with a variable number of devices while keeping secure connections and maintaining reasonable response times.
- Hardware and software evolution: the strength of a security algorithm can be compromised by two main factors. One is the computational capabilities of processors growing at a fast pace. The second factor is that new software solutions may speed up the breaking of a security algorithm. Because of this fact, the security mechanisms chosen must allow for increasing the security level at any time while maintaining reduced data storage and computational needs.

In an IoT fog computing scenario, Transport Layer Security (TLS) arises as one of the best positioned candidates, but it has the problem that most popular standard ciphering suites available were not designed having in mind the limitations of resource-constrained and battery-operated devices. This fact has been changing in the last years, since lighter and more future-proof alternatives are being supported and implemented widely by the standard.

This article includes three main contributions aimed at fostering security in resource-constrained energy-efficient IoT fog computing gateways. First, it presents a detailed review of the main and the latest security concerns in IoT systems. Such a review is completed with a clear description of the most used mechanism for securing Internet communications, TLS. Moreover, several comparative studies of Elliptic Curve Cryptography (ECC) and Rivest–Shamir–Adleman (RSA) in terms of performance and energy efficiency are analyzed. Second, a series of tests are conducted to determine the performance and power-consumption impact of using TLS over plain HTTP communications and to measure the differences between RSA and ECC in a real-world scenario in terms of security, scalability, power consumption, and data throughput when implemented in an IoT network. Third, the RSA results are then compared to those obtained by a more suitable approach for IoT gateways, consisting of using ECC algorithms with key sizes that guarantee an acceptable security level for years to come.

The rest of this article is organized as follows. Section 2 enumerates the main state-of-the-art hardware platforms and security concerns in IoT fog computing systems, describes the basics of TLS, and compares RSA and ECC in terms of complexity and performance. Section 3 describes the hardware and software of the testbed designed. In Section 4, multiple experiments are conducted and their results are analyzed. Finally, Section 5 is devoted to conclusions.

## 2. Related Work

### 2.1. Performance and Security of IoT Hardware for Resource-Constrained Energy-Efficient Gateways

The number of IoT hardware platforms that can be integrated as gateways in a fog computing system has grown from a few alternatives to a large and heterogeneous ecosystem. In recent years, the evolution of embedded hardware power efficiency and integration has allowed IoT development boards to achieve computer-like computational capabilities. This fast evolution makes it very difficult to give a precise definition of resource-constrained devices in terms of memory size or processor clock rate. One definition of constrained-node networks can be found in [28]. Three levels of constrained devices are defined attending at RAM and flash memory available. Because of the fast evolution of embedded hardware capabilities, this classification fails to give an updated definition of constrained device. As it can be seen in Table 1, even the less powerful boards available nowadays greatly exceed the memory values defined by each of the three classes. Some of the boards presented in the table are also analyzed in [29], where an overview of power efficient development boards for IoT applications is presented.

As it was mentioned in Section 1, security in IoT systems is often overlooked or addressed lightly by using weak or ad hoc approaches. For instance, in the case of [23], although TLS tests are performed, a Pre-Shared Key (PSK) cipher suite (i.e., PSK-AES-128-CCM-8) is used. Since only symmetric key operations are used for authentication [30], it does not require as much memory as asymmetric cipher suites. The use of this type of cipher suites constrains the devices to a very specific scenario (e.g., since PSK is used, it has to be established manually and set up in advance). On top of that, this cipher suite is also considered insecure and not recommended [31].

A comparison of the computational capabilities of some of the latest hardware platforms available to implement secure resource-constrained energy-efficient IoT gateways is presented in Table 1. The table shows the name of the board, the clock rate along with the number of cores of the main processor, the secondary processor (if available), the amount of embedded RAM and use references.

### 2.2. IoT Security

The IoT paradigm raises public security concerns, including personal privacy issues, threats of cyber-attacks, and organized crime [45]. In order to confront the uniqueness of IoT in terms of the upcoming security challenges, several approaches have been proposed in the literature. For instance, Vasilomanolakis et al. [46] devised a comprehensive list of privacy and security requirements with the aim of establishing a standard set of security specifications for IoT technologies. Moreover, Atamli et al. [47] introduced a threat model as a method to analyze the impact of threats in different applications. From such a threat model, the authors deduce the security and privacy requirements.

Leo et al. [48] focused on the design of an architecture for secure exchanges in IoT services. Such researchers proposed an architecture that is mainly devoted to deploying and managing a federated environment for an authority delegation mechanism, identity-based capabilities, and dynamic context information. Another piece of architecture for supporting privacy and security in IoT systems is presented in [49].

Numerous vulnerabilities have also been detected in IoT communication technologies, middleware, and Machine-to-Machine (M2M) communications. For example, Grabovica et al. [50] explored the security protocols provided by communication technologies like Radio-Frequency IDentification(RFID), Bluetooth, Wi-Fi, and ZigBee. Furthermore, Ngu et al. [51] presented a survey on the capabilities and challenges of IoT middleware architectures ranging from cloud-based, service-based and actor-based architectures. A cloud-based IoT middleware architecture is limited for what is available on the cloud and it varies widely among the different cloud-based platforms. Typically, their functionalities are exposed as a set of Application Programming Interfaces (APIs). The functionalities provided can be as simple as a high performance storage system or a powerful computation engine with predefined monitoring and analysis tools. In the case of a service-based architecture, a high-performing middleware is generally deployed on multiple nodes running in the cloud or on powerful gateways between IoT devices and the applications. It is not designed to be deployed in resource-constrained IoT devices and does not support device-to-device communications. In an actor-based architecture, the middleware is designed to be lightweight and can be embedded into all the layers (i.e., sensory layer, mobile access layer, and the cloud). The basic middleware computation units are thus distributed in the network. It provides the best latency and scalability for large-scale connected IoT devices. Regarding M2M communications, Barki et al. [52] provided a survey that addresses the security challenges and threats that arise when dealing with a fusion of heterogeneous networks.

Furthermore, other researchers suggested security enhancements that rely on a layer-based approach [53]. An exhaustive analysis on the security protocols and mechanisms available, together with its operational layer and the security properties and functionality supported, is presented in [54].

In addition, it is worth mentioning that security based on random physical media and objects is a fast-growing field. The unique and unclonable character of disordered physical structures can be exploited to address many vulnerabilities. A classification of past and ongoing work in physical disorder-based security along with security analyses and implementation examples is given in [55]. To enable end-to-end security in constrained network environments, some researchers focused on different IP-based security protocols for IoT, like Datagram TLS (DTLS) [56], the HIP Diet EXchange (DEX), and minimal IKEv2. For instance, Hummen et al. [57] identified the challenges that arise when employing such protocols and provide a high-level overview of approaches proposed to counteract the design-level protocol issues identified. Moreover, Abeele et al. [58] present an approach that relies on a trusted gateway to mitigate the overhead of the DTLS handshake in IP-based networks, providing the flexibility needed to support a variety of security requirements.

### 2.3. Securing TCP/IP Communications: TLS

Most IoT deployments, such as the home automation system evaluated in [13], are based on TCP/IP communications. In order to secure the data transmission in the auto-configuration and auto-registration protocol, along with the subsequent transducer access, the best option is to use the TLS [59] standard. For systems that do not depend on TCP/IP, another option could be to use DTLS, the TLS alternative for User Datagram Protocol (UDP)-based protocols. It was developed for covering the security needs of new protocols, such as Constrained Application Protocol (CoAP), which uses UDP instead of TCP. CoAP is basically a reduced HTTP implementation designed to work over 6LoWPAN networks for IoT applications [54]. CoAP’s main objective is to minimize the overhead created by the non-payload data required by HTTP. One problem of this approach is that UDP communications are unreliable. To solve this issue, CoAP defines a reliability mechanism based on request-response messages that acts as a lightweight TCP implementation using UDP. CoAP was designed having resource-constrained devices in mind, so security is addressed lightly, with a lot of limitations, such as the use of pre-shared keys or raw public key cipher suites [60]. Certificates can also be used, but the cipher suites supported have low security levels. Furthermore, Sehgal et al. [23] concluded that DTLS uses more RAM and stack memory than TLS, meaning that, for the same security level, there is no advantage on using DTLS instead of TLS. Hence, only TLS is considered for further analysis.

The primary goal of TLS is to provide privacy and data integrity to the communications performed over the Internet. One advantage of TLS is that it is independent from the application protocol. Higher-level protocols can layer on top of the TLS protocol transparently. For instance, the commonly used HTTPS [61], the protocol to secure Internet web page access and transactions, uses TLS to secure the communications channel. A large set of protocols, such as File Transfer Protocol over SSL (FTPS), Simple Mail Transfer Protocol Secure (SMTPS), Real Time Streaming Protocol (RTSP), Internet Message Access Protocol over SSL (IMAPS) and many more, rely on TLS.

The main goals of the TLS protocol are the following:

- Cryptographic security: TLS can be used to establish a secure connection between two parties.
- Interoperability: two different programmers should be able to develop applications that can establish a secure communication successfully, without any knowledge of each other’s code, making use of TLS.
- Extensibility: TLS seeks to provide a framework into which new public key and encryption methods can be incorporated. This removes the need for new protocol creation or entire library implementations, reducing the risk of introducing new weaknesses.
- Relative efficiency: cryptographic operations tend to be Central Processing Unit (CPU) intensive, particularly public key operations. TLS aims to be as efficient as possible while achieving the security level required.

The protocol is composed of two layers: the TLS Record Protocol and the TLS Handshake Protocol. The TLS Record Protocol provides connection security with two basic properties:

- Connection privacy: communications are encrypted by using symmetric cryptography (e.g., AES, RC4). The symmetric keys involved are generated uniquely for each connection and are based on a secret negotiated previously by another protocol (e.g., the TLS Handshake Protocol).
- Connection reliability: The message transport includes message integrity and authenticity checks using a keyed Message Authentication Code (MAC). This is achieved by using secure hash functions (e.g., SHA-1) for MAC computations.

The TLS Handshake Protocol allows server and client to authenticate each other and to negotiate an encryption algorithm along with the cryptographic keys needed for the application protocol to transmit or receive data. The three basic properties that the TLS Handshake Protocol grants are:

- The peers involved are authenticated using public key (i.e., asymmetric) cryptography (e.g., RSA, Digital Signature Algorithm (DSA)).
- It is secure to negotiate a shared secret. As it was mentioned before, the TLS Record Protocol uses symmetric key cryptography. The shared secret can be negotiated whilst remaining unavailable to eavesdroppers and, for an authenticated connection, the secret cannot be obtained, even using a Man-in-the-Middle (MitM) attack.
- The negotiation is reliable: it is not possible for an attacker to modify the negotiation communications without being detected by the parties involved in such communications.

The TLS Handshake Protocol has three subprotocols that are used to allow peers to agree upon security parameters for the record layer (i.e., the cipher suite to be used), to authenticate themselves, to instantiate the security parameters negotiated, and to report error conditions to each other.

### 2.4. TLS Handshake Procedure and TLS Cipher Suites

The TLS Record Protocol is the one in charge of securing the connection once the TLS Handshake Protocol takes place and after agreeing on the parameters to use during the TLS session.

A cipher suite is defined by its name, which indicates the algorithms involved in both the TLS Handshake and the TLS Record operations. For example, the cipher suite ECDHE-RSA-AES128-GCM-SHA256 uses:

- ECDHE-RSA: Elliptic curve Diffie–Hellman Ephemeral (ECDHE) with RSA signing for the key-exchange algorithm.
- AES128-GCM: Advanced Encryption Standard (AES) with a key length of 128 bits as the block cipher. GCM stands for Galois/Counter Mode and defines the mode of operation of the symmetric key block cipher. AES is a symmetric encryption algorithm, and the secret used to derive the ciphering key is the one obtained by the previous use of ECDHE-RSA during the TLS Handshake.
- SHA256: Secure Hash Algorithm with a hash result of 256 bits. This is used as the Pseudo-random Function (PRF) to ensure the cryptographic integrity of the handshake messages.

In the case of ECC, a more detailed explanation of its cipher suites can be found in [62].

There are two main subgroups of cipher suites recommended for TLS [31]: RSA and ECDHE. RSA-based cipher suites use RSA as the key-exchange algorithm, while the ECDHE-based ones use an algorithm that makes use of Ephemeral Diffie–Hellman based on Elliptic Curves. The Ephemeral part means that the key is regenerated after each session, providing Perfect Forward Secrecy (PFS) in contrast to the variants based on RSA.

**Figure 2:**
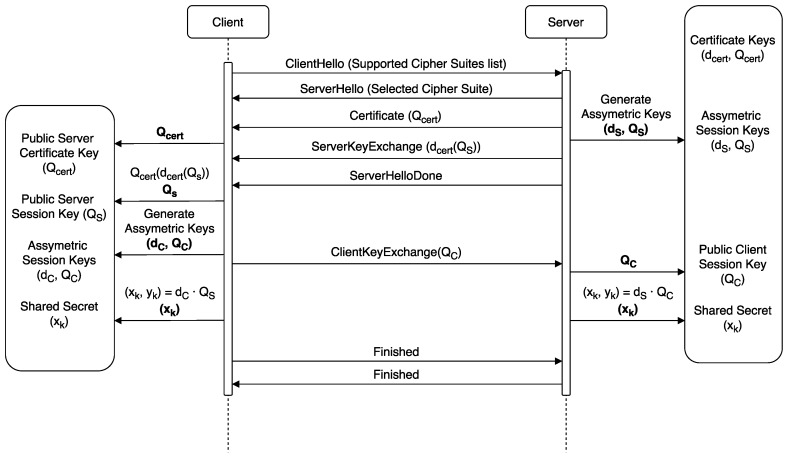
Transport Layer Security (TLS) Handshake procedure for ECDHE-RSA-AES128-GCM-SHA256 and similar cipher suites.

Figure 2 illustrates the messages exchanged during the handshake when using the cipher suite with a ECDHE key-exchange algorithm. $d_{x}$ refers to private keys, while public keys are the ones defined as $Q_{x}$. The procedure takes place as follows:

- The client sends a ClientHello specifying its supported cipher suites.
- The server responds with a ServerHello with the cipher suite selected. This is the cipher suite that is going to be used during the whole TLS session.
- The server sends its certificate in a Certificate message. Along with it, the public key ($Q_{cert}$) of the aforementioned certificate is sent.
- The server generates a key pair ($d_{s},Q_{s}$) needed for the ECDHE algorithm and sends the public key to the client, encrypted with the private key of the certificate ($d_{cert}\left(Q_{s}\right)$). This corresponds to the ServerKeyExchange message.
- Once the client receives the ServerKeyExchange, it uses the certificate’s public key received in the Certificate message to check the authenticity of the ECDHE public key by verifying the RSA signature ($Q_{cert}\left(d_{cert}\left(Q_{s}\right)\right)$), thus obtaining the ECDHE public key ($Q_{s}$) of the server.
- Finally, the client generates its own ECDHE key pair ($d_{c},Q_{c}$) and sends the public key to the server.
- At this point, both server and client can obtain the Session Secret by performing an operation (ECC dot product) with one’s own private key and the other party’s public-key. For instance, the client will perform the dot product of its own private key and the server public key ($d_{c}Q_{s}$) obtaining as a result a the coordinates of an elliptic curve point ($x_{k},y_{k}$). The *x*-coordinate of this point is then used to generate the session key needed for the AES128-GCM block cipher, by using a Key Derivation Function (KDF) [63]. The client sends a finished message encrypted using AES-128 and the server responds with an equivalent message.

It is important to note that many connections can be established using the same session through the resumption feature of the TLS Handshake Protocol, but eventually sessions must be renegotiated. Moreover, it is not always possible to accommodate the use of TLS resumption on IoT fog gateways, and, depending on the number and periodicity of the connections, its benefits can be limited.

### 2.5. Cipher Suites for IoT Fog Computing Applications

As explained in Section 2.4, ECDHE can be used as the key-exchange algorithm to obtain PFS. Note that DHE can also be used, but it is clearly outperformed by ECDHE [64]. The only reason to use DHE instead of ECDHE is the existence of possible incompatibility issues, but nearly every TLS implementation supports both algorithms [65,66,67,68]. ECDHE-RSA cipher suites signs the ServerKeyExchange message using the RSA public key certificate. In addition, certificates can be generated with different public key sizes, 2048-bit RSA being the minimum size considered secure nowadays. In addition, 768-bit RSA was factored in 2010 using the number field sieve factoring method [69] and a 1024-bit RSA implementation of OpenSSL was successfully broken using a fault-based security attack in less than 100 h [70]. However, note that the use of a 2048-bit certificate on an ephemeral key-exchange algorithm introduces heavy overhead and computing requirements, which are very difficult to accommodate on the constrained hardware capabilities of most IoT devices. The encryption and decryption processes take place every time a device accesses or sends data over a secure connection. Although expensive public-key operations are needed only in the beginning of the communications (i.e., during the TLS Handshake), they are renegotiated when a new session is established. Moreover, IoT gateways, that need to manage a great number of connections are even more affected by this encryption overhead, reducing throughput, and increasing power consumption.

#### 2.5.1. Public-Key Security Levels

In order to understand better why RSA needs such a big key size to be considered secure, this section gives an overview of how public-key sizes relate to security.

Public key cryptography is based on the theoretical robustness of trapdoor functions. A trapdoor function is a mathematical expression that can be easily computed in one direction, but it is very difficult to compute in the inverse way without some specific information, which is called the trapdoor. The security level of a public-key cryptosystem is a way of measuring the effort to break its trapdoor function. The effort is generally expressed as the number of operations needed to break the cryptographic primitive, meaning that a k-bit security level will need $2^{k}$ operations to be broken and, thus, it offers a security level *k*. With symmetric cryptosystems, the key-length selected directly relates to the security level offered by the cryptosystem. However, when applying this concept to public-key schemes, it is not easy to give a value for the security level relying on the length of the key.

For example, the RSA trapdoor function is based on the assumption that factorizing large integer numbers that are the result of two large prime numbers is a difficult problem to solve. If the prime numbers used are big enough, the resultant encryption is considered secure.

**Figure 3:**
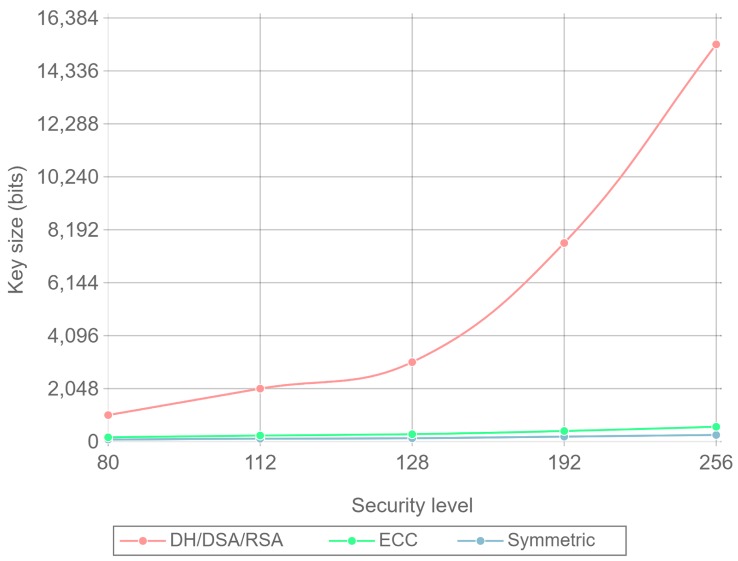
Key size needed for different security levels using symmetric, Rivest–Shamir–Adleman (RSA) and Elliptic Curve Cryptography (ECC) ciphers.

The process of finding the required prime numbers is fairly simple and the operations required are not computationally expensive. The problem with the RSA system is that existing algorithms such as Quadratic Sieve [71] or General Number Field Sieve [72] allow for a faster integer factorization than brute force or prime guessing approximations. The problem is even worse, due to the fact that these algorithms work much better the larger the number they are trying to factorize. As a consequence, the security level that RSA can provide does not grow lineally with key size. This behavior can be observed in Figure 3, where RSA, ECC, and symmetric ciphers are compared. The key size needed for the different security levels presented is described in Table 2 [73]. The actual values shown in Figure 3 are taken from TLS curve implementations [62] in the range of these key size values. It can be observed that even for low security levels, RSA key sizes are much larger than ECC key sizes.

Note that there is not a direct correspondence between security levels and key sizes. For instance, as described in Table 2, for a 112 security level, a 2048-bit key size must be used if an RSA cipher is selected, but only a 224-bit key size is needed if the cipher uses ECC. Moreover, these differences increase with the security level. A comprehensive explanation of security levels, why they can be misleading and how they are obtained for each type of cipher can be found in [74].

As a final conclusion, it can be stated that security levels offer a much better understanding of how secure a given algorithm is instead of only relying on the key-size used. In the following sections, the values presented in Table 2 for ECC and RSA are considered as the reference of the security provided.

#### 2.5.2. ECC vs. RSA: Power Consumption and Performance

Until today, techniques like RSA and ECC were not used in IoT nodes because they have resource demanding requirements. Thus, other alternatives have been studied. For example, research work in key management schemes has been conducted in [75,76]. Mbarek et al. [77] compared and analyzed two different authentication protocols: Security Protocols for sensor Networks (SPINS) and TinySec. For such an analysis, the authors made use of the Network Simulator version 2 (NS-2) to compare the performance of both authentication protocols.

Recent research has been focused on the usage of RSA and ECC for resource-constrained devices and studied the basic operations of Elliptic Curve Digital Signature Algorithm (ECDSA) and Elliptic curve Diffie–Hellman (ECDH) [78]. For instance, in [79], both algorithms are analyzed, and the application of ECC is recommended to increase security and speed. However, the authors remark that, in order to maximize the performance in chips, the ECC implementation would need consistent enhancements.

A time performance comparison between ECC (secp160r1, secp192r1, secp224r1) and RSA (1024 and 2048 bits) is conducted in [80] using 8-bit CPUs. In such a paper, the researchers describe the implementation of the algorithms and present execution times and detailed information about the more time-consuming operations. The authors conclude that ECC outperforms RSA for the key sizes tested, and that it would be even more efficient for larger key sizes.

Other authors studied the communications overhead that a TLS handshake requires when used in conjunction with ECC [81]. The experiments presented focused on the number of messages exchanged by third-party cipher suites and by their own ECC library for embedded systems. Their results represent a useful guide when considering a trade-off between security and performance in resource-constrained scenarios.

ECC and RSA power consumption has been also studied in different scenarios [24,82,83]. Moreover, a framework for analyzing power consumption of cryptographic algorithms and security protocols is proposed in [84]. In such a paper, the authors examine several cryptographic algorithms from the three main classes (i.e., asymmetric, symmetric, and hash) jointly with a comprehensive analysis of the energy requirements of the security mechanisms. They also study the power consumption requirements of the transport-layer security protocol (Secure Socket Layer (SSL)) considering the impact of various parameters at the protocol level (such as cipher suites, authentication mechanisms, and transaction sizes) and the cryptographic algorithm level (i.e., cipher modes, strength). The researchers carried out the evaluation on a Compaq iPAQ H3670 Pocket PC, which contains an Intel SA-1110 with a Strong ARM processor clocked at 206 MHz, 64 MB of RAM, and 16 MB of Flash ROM. The paper presents the power consumption of the different steps involved in the algorithms analyzed (e.g., key generation, key exchange, signing, verifying). They also tested the SSL protocol to transmit payloads that range from 1 KB to 1 MB. After the analysis, the authors presented five conclusions. First, asymmetric and hash algorithms have the highest and least energy costs, respectively. Second, the energy cost of asymmetric algorithms is dependent on key size, while symmetric algorithms are not significantly affected. Third, the power consumption of a symmetric algorithm depends not only on the bulk data encryption and decryption cost, but also on the key set-up cost. Fourth, wide variations in power consumption exist within the same family of cryptographic algorithms, and finally, a trade-off between the level of security provided by the algorithm and energy savings can be achieved by tuning the parameters.

All the works mentioned provide interesting conclusions and insight into the ECC versus RSA dilemma. Power consumption and performance results comparing both algorithms are presented, but all of them fall short at some point. Not taking into account security levels, comparing the algorithms with an ad hoc implementation and in an isolated way, providing an approximated value of the energy consumption or not measuring it with external devices, or the use of hardware platforms that do not provide mid-term valid results are some of the drawbacks found. Furthermore, some references do not use real communication protocols or use insecure key-lengths or deprecated cipher suites. None of them compares together power consumption and throughput for real-life IoT fog computing scenarios with adequate security levels.

## 3. Evaluating Power Consumption and Throughput in Resource-Constrained IoT Gateways

In this section, a series of tests are conducted to evaluate the impact of TLS on IoT communications performed by IoT gateways. Since the selected IoT gateways have to cope with constrained resources (i.e., they require reduced power consumption and have limited computing power) and they are usually deployed for extended periods of time, cryptographic schemes should be designed to be efficient, lightweight, and robust.

The objective is to determine the performance and power-consumption impact of using TLS over plain HTTP communications, and measure the differences between RSA and ECC in a real-world scenario. Note that the use of HTTP is due to the fact that a fog abstraction layer hides the platform heterogeneity by providing generic APIs [85], which are usually implemented by using plain HTTP or HTTP-like APIs that are accessed through horizontal and vertical links (i.e., by performing intra-fog and inter-fog communications).

### 3.1. Hardware Testbed

The hardware testbed proposed needs to meet two major objectives. First, it has to support the cipher suites selected in Section 3.4 and allow for using well-known and tested implementations of TLS. The usage of specific hardware with ad hoc TLS implementations could bias the results due to the ciphering algorithm implementations. Therefore, by using some open-source and extensively used TLS implementation such as OpenSSL [86], the performance and energy consumption differences between ciphering algorithms should be as close as possible across different hardware and software platforms. Second, in order to provide medium- and long-term valid conclusions, the selected hardware must fulfill future performance expectations for energy-efficient IoT gateways.

To comply with all these requirements, it was decided to use a Single Board Computer (SBC) based on a low power consumption SoC. The Cortex-A7 [87] was chosen since it fits the requirements and it is the most efficient ARMv7-A processor and the most commercially successful with more than a billion units in production.

**Figure 4:**
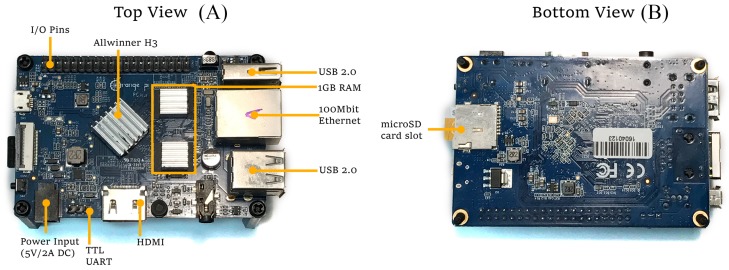
Orange Pi PC Printed Circuit Board (PCB). Top view (**A**) and bottom view (**B**).

Note also that three SBCs are actually needed: one running as server, another running as client, and a third in charge of measuring power consumption. Several SBCs were evaluated and Orange Pi PCs [88] were finally selected, since they offer a good trade-off between features and cost (as of writing, its price starts as low as $15). This SBC uses an Allwinner H3 SoC [89] with a quad-core Cortex-A7 configuration. The detailed PCB layout of an Orange Pi PC is depicted in Figure 4. Using a different SBC or SoC-based platform could yield different total power consumption results, but the conclusions obtained when comparing cipher suites should not change.

It is worth mentioning that, during the tests, in order to measure power consumption, external hardware was used instead of the Advanced Configuration and Power Interface (ACPI) of the SBCs. In this way, current readings are independent from the particular ACPI-related hardware of the SBC and the setup remains valid for any other SBC.

The main characteristics of the Orange Pi PC are:

- 1 GB DDR RAM.
- Allwinner H3 SoC, A7 quad-core 1.6 GHz processor.
- 100 Mbit Ethernet.
- 3 USB 2.0 ports.
- 5 V/2 A power input.
- HDMI output.

Although the Allwinner H3 SoC has hardware-accelerated encryption support powered by a Crypto Engine (CE), it was not used during the tests, since it can distort the results obtained. Moreover, it must be mentioned that, as of writing, the driver available for the CE does not support RSA or ECDSA acceleration [90].

During the experiments, the Orange Pi PCs were connected using a TP-Link TL-SG108 Gigabit switch and ad hoc Cat 5e Ethernet patch cables. To power up the system, a fixed 5 V/12 A power source was used. The SD cards used were four 32 GB Samsung Evo MB-MP32DA/EU cards.

**Figure 5:**
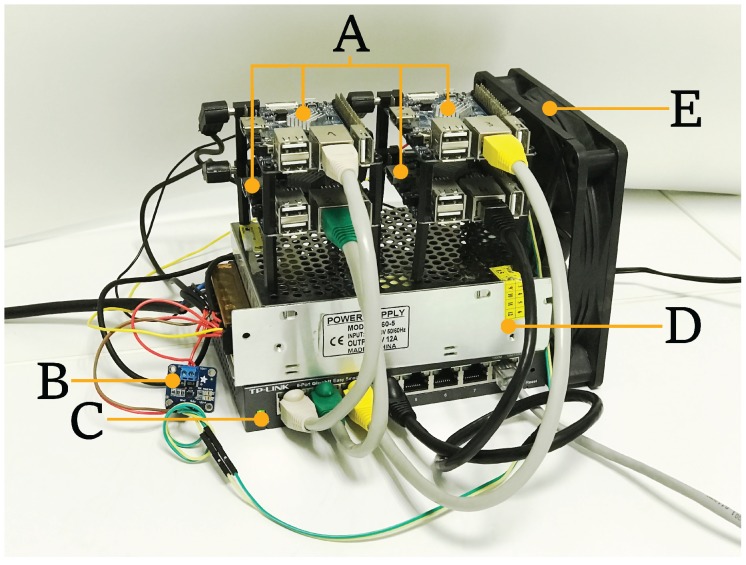
Testbed. (**A**) Orange Pi PC Single Board Computers (SBCs); (**B**) INA219; (**C**) switch; (**D**) power supply; (**E**) cooling fan.

To measure the current being drawn, an Adafruit INA219 was selected, since it offers enough precision (it can be configured in high precision mode measuring 0.1 mA steps with a maximum of ±400 mA or in low precision mode measuring 0.8 mA steps and a maximum of ±3.2 A), and allows for measuring up to 26 V. The Inter-Integrated Circuit I2C bus used by the INA219 makes it easy to configure the device and to measure values in an automatized way. For instance, an INA219 was utilized by GreenMiner [91], a framework aimed at measuring the real energy consumption of a given application running in an actual smartphone. Figure 5 shows the four SBCs (three used for the experiments and one used as a gateway to allow external access to the testbed) along with the INA219, the switch, the power supply, and one fan for cooling purposes.

### 3.2. Testbed Architecture

**Figure 6:**
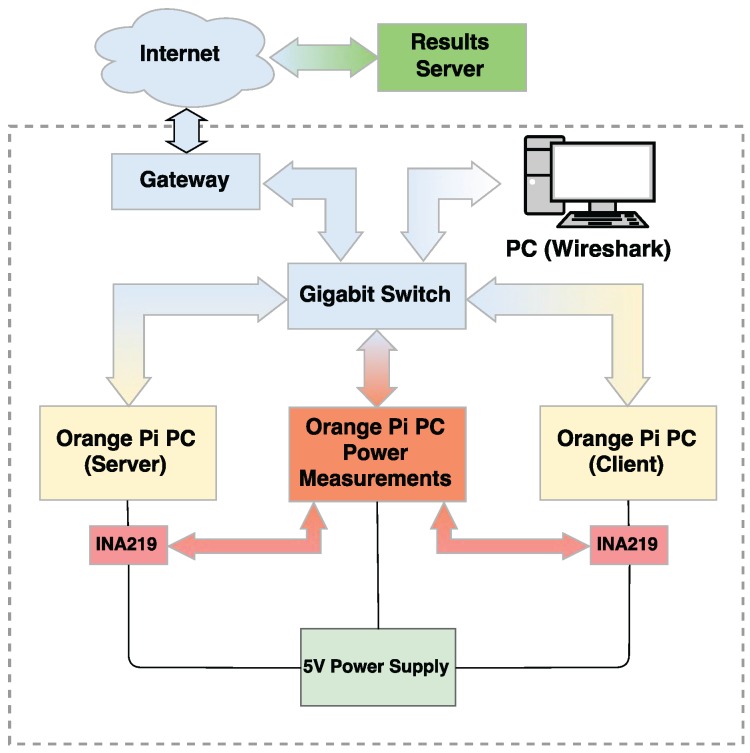
Detailed testbed architecture.

The different hardware components described in the previous subsection are organized in the architecture described in Figure 6. The 5 Volt supply powers directly the Orange Pi PC in charge of acquiring the energy consumption samples. Between the power supply and the other two Orange Pi PCs (one acting as a server and the other acting as a client), two INA219 modules are installed, one for each PC. The INA219 modules are also connected to the third Orange Pi PC using the I2C bus, allowing it for accessing the modules and reading the desired values. A Gigabit switch is in charge of providing network connectivity to the three SBCs. A PC with Wireshark installed is also connected to the switch, in order to perform a frame time analysis. Finally, the Gigabit switch is connected to the Internet using a domestic Gateway (do not confuse with the concept of IoT gateway), in order to provide Internet access to the Orange Pi PC in charge of acquiring the energy consumption samples. The Internet connectivity is only needed for simplifying the process of extracting the values obtained during the tests, so they can be processed and analyzed. In this way, the results are uploaded to a server where they can be easily accessed.

### 3.3. Software

The operating system installed on the Orange Pi PC was ARMbian [92], a modified Debian distribution for ARM-powered devices.

The HTTP servers used for the tests were Apache2 and Nginx. These two web servers were chosen since they are, as of writing, the most popular [93], with a market share of 51.5% and 31.3%, respectively. During the experiments, the same tests were run in two different web servers to eliminate any bias that could be introduced by the server implementation or configuration. In addition, both servers used the same TLS library (OpenSSL).

To automate the HTTP/HTTPS request generation process, an HTTP benchmark software was used. Some alternatives were studied, such as Apache Benchmark [94], Httperf [95] or Siege [96]. Eventually, Siege was selected because, although significant differences were not found among the different benchmark software in terms of features, Siege was easier to run and parse, and presented all the necessary configuration parameters needed for the tests.

The configuration used for Apache2 and Nginx was left to default values. Both servers were installed using Aptitude and the default repositories of the ARMBian distribution. It was verified that both servers were configured for using all four logical processors available on the Orange Pi PC SOCs. The only change in configuration was made to enable and disable GNU ZIP (GZIP) compression and configure the same compression level on both Apache2 and Nginx when compression was used. A compression level of 6 was selected, since it provides a good trade-off between computational cost and size reduction on the resulting compressed data.

The specific versions of the software used were:

- ARMBIAN Debian GNU/Linux 8 (jessie) 3.4.112-sun8i / #10 SMP PREEMPT Sun Oct 23 16:06:55 CEST 2016 armv7l.
- Apache/2.4.10 (Debian) Sep 16 2016 10:04:38.
- nginx/1.6.2.
- OpenSSL 1.0.1t May 3rd 2016.
- SIEGE 3.0.8.

### 3.4. Selected Cipher Suites and Certificate Generation

The main algorithms involved in a cipher suite were examined previously in Section 2.4. The public key authentication, where the signing and verifying processes are performed during the TLS handshake, demands the highest computational power. To analyze the impact of ECC and RSA, two identical cipher suites were selected, with the only difference being the signing algorithm used during the key exchange (i.e., RSA and ECDSA):

- ECDHE-RSA-AES128-GCM-SHA256.
- ECDHE-ECDSA-AES128-GCM-SHA256.

It is important to note that both cipher suites use ECC in the key-exchange process (i.e., ECDHE), but the key signing and verifying processes are carried out using RSA and ECDSA, respectively. Both cipher suites chosen are among the few recommended by the National Institute of Standards and Technology (NIST) guidelines for cipher suite selection and configuration [31], sharing all the parameters but the signing algorithm.

As explained in [62], ECDHE_ECDSA cipher suite certificates must contain an ECDSA-capable public key and the ServerKeyExchange parameters must be signed with this key. Equivalently, ECDHE_RSA cipher suite certificates must contain an RSA-capable public key. Thus, two different certificates are needed, one for each cipher suite.

OpenSSL was used in order to generate the certificates. The selected key sizes for each certificate are obtained from Table 2, for a strength level of 112. For RSA, a 2048-bit key size was selected, and, for ECC, the secp256r1 curve (also known asprime256v1 andNIST P-256) was chosen. Such a curve corresponds to a 256-bit ECC key size; therefore, the ECC certificate is closer to a strength level of 128 than to a 112 strength level. This difference in strength level obeys for two reasons. First, 2048-bit RSA is one of the most used signing systems for TLS and the curve *secp256r1* is one of the TLS ECC curves with greater support. Second, the experiments were designed to show the advantages of ECC over RSA, so putting ECC in a worse scenario than RSA in terms of performance should display the benefits of ECC.

## 4. Experiments

### 4.1. Testbed Setup

To evaluate the performance differences between the selected RSA and ECDSA cipher suites, a series of tests were performed. A set of files using JSON data were created using lorem ipsum extracts [97], along with randomly generated strings to obtain the desired file sizes. JSON data files were generated through a Python script using the library Faker (version 0.7.3) [98]. This file generation process was conceived with two goals in mind: to allow for creating files with the exactly desired size, and to make use of data structures similar to the ones usually transmitted by IoT devices, achieving GZIP compression ratios similar to real scenarios. An analysis on the compression ratios that can be reached for different types of data can be found in [99]. The sizes of the files generated ranged from 32 bytes to 131,072 bytes (128 kilobytes) in a base-2 exponential progression. The *x*-axis spacing of the charts presented in this section correspond to the value log2(bytes), but the byte values are presented instead of the logarithmic value for a more intuitive representation.

The impact of the signing algorithm used in the TLS handshake (i.e., ECDSA or RSA for the cipher suites selected) is independent of the size of the payload transmitted by the underlying HTTP communications. Since the aim of the tests performed was to determine the relative impact of the ciphering algorithm in a real IoT scenario, payloads with different sizes were used. Typical payloads transmitted by IoT gateways when managing requests from a few IoT nodes are represented with the lower values used in these tests (i.e., from 32 to 1024 bytes). Nevertheless, IoT gateways can transmit larger payloads as a result of carrying out data aggregation for several IoT nodes. Payloads as large as 128 kilobytes are used to determine whether input/output operations and network transactions have an impact on power consumption and data throughput when using different cipher suites. These tests allow not only determining the suitability of ECDSA over RSA for small fog computing networks, but also for densely populated IoT networks.

For each file size, Siege was run making use of the multiple configuration parameters it offers, which allow for varying the number of concurrent clients, the amount of requests per client, the delay between requests, and much more. The configuration used was as follows:

- Benchmark mode: no delay between client requests.
- 200 requests per client.
- Concurrent clients from 2 to 128 in a base-2 exponential progression.

Combining the file sizes with the number of concurrent Siege clients, a total of 91 separate tests were performed for each cipher suite and web server. The same tests were also conducted for plain HTTP to determine a reference baseline where no encryption algorithms were used. All tests were run using both GZIP compression and no compression at all. Thus, Siege was run 1092 separate times to obtain the results presented. The same tests were performed two times, one for measuring the server side power consumption and another to measure the client side.

### 4.2. Baseline Power Consumption Test

In order to analyze the impact of the hardware platform and the Linux distribution on the power consumption, several tests were performed. For an interval of ten minutes, an instantaneous power consumption sample was obtained each second for three different scenarios.

- Idle state: the Orange Pi PC was running the Linux system while the Nginx web server was left in an idle state, with all the other services running.
- Load state: using another Orange Pi PC board, Siege was executed with 16 concurrent clients against the Orange Pi PC while running the Nginx server. This test was performed by downloading a 512 byte file with Siege configured in benchmark mode. This test was performed using two different configurations:
  – HTTPS and Nginx configured to use the ECDSA cipher suite.
  – Plain HTTP.

**Figure 7:**
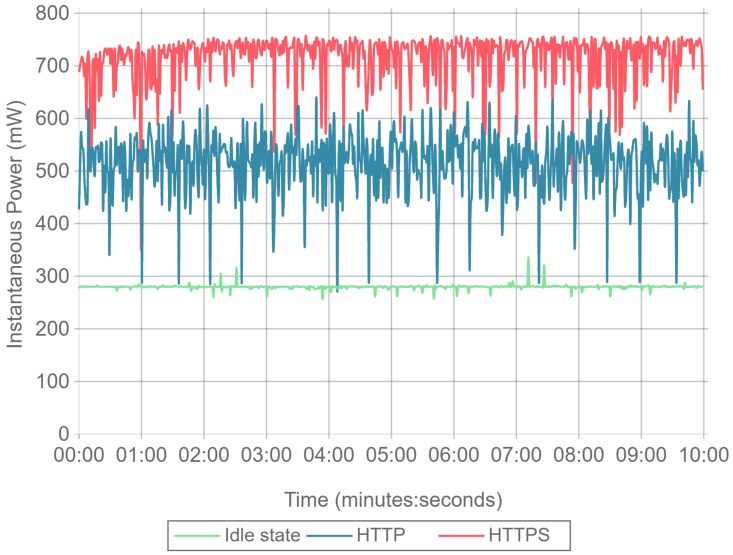
Power consumption during 10 min for idle state and 16 concurrent clients accessing a 512 byte payload served by Nginx using HTTPS and HTTP.

With these tests, three different power consumption baselines were obtained, as it is illustrated in Figure 7. 

As it can be observed, power consumption remains stable when the board is idling, with minimal and low amplitude deviations. When using HTTP, power consumption increases, but it presents several drops that even reach idle power consumption values. This is explained due to the fact that the HTTP requests are processed extremely fast, leading to very short time intervals where no input/output or network operations are being carried out by the Nginx server. As expected, HTTPS power consumption is the highest and it presents less amplitude variation. Compared to plain HTTP, the use of TLS is more CPU intensive and leads to longer lasting transactions that keep the CPU from idling.

After analyzing these results, it can be concluded that the hardware and software of the testbed has a very stable base power consumption. Furthermore, it can be stated that any relevant energy consumption differences observed during the next tests can be attributed directly to the cipher suite algorithms evaluated.

### 4.3. Analysis of the Effect of Compression, Server Implementation, and Cipher Suite Selection on Power Consumption and Data Throughput

**Figure 8:**
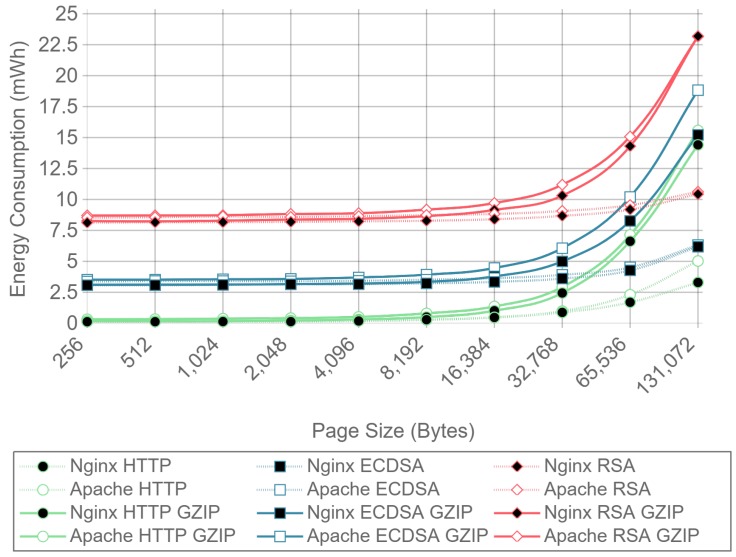
Server-side page size versus energy consumption for Apache2 and Nginx with 16 concurrent clients, with and without GNU ZIP (GZIP) compression.

Figure 8 shows the server side energy consumption values for both Apache and Nginx running the ECC and RSA cipher suites, as well as for plain HTTP communications. Both GZIP compression (solid lines) and no compression (dotted lines) test results are represented, using all the different payload size files except the ones for which compression causes larger payloads to be transmitted due to GZIP overhead (32, 64, and 128 bytes). For the sake of clarity, only the 16 concurrent client test charts are shown. As it can be observed in the next figures, the results with this number of clients are more stable than the ones obtained with less concurrency. By using more concurrent clients, the absolute energy consumption value varies, but the differences between implementations remain constant. As it can be seen, there is almost no difference in energy consumption for each curve until the payload size exceeds 4096 bytes. With 4096 bytes or more, all six combinations of cipher suite and web server present an increment on energy consumption when using GZIP compared with using no compression. This is due to the fact that the compression of large files takes more time and power, and in a general IoT scenario, the gains of sending less data through the network do not compensate for the effort of compressing data in terms of total energy consumption.

**Figure 9:**
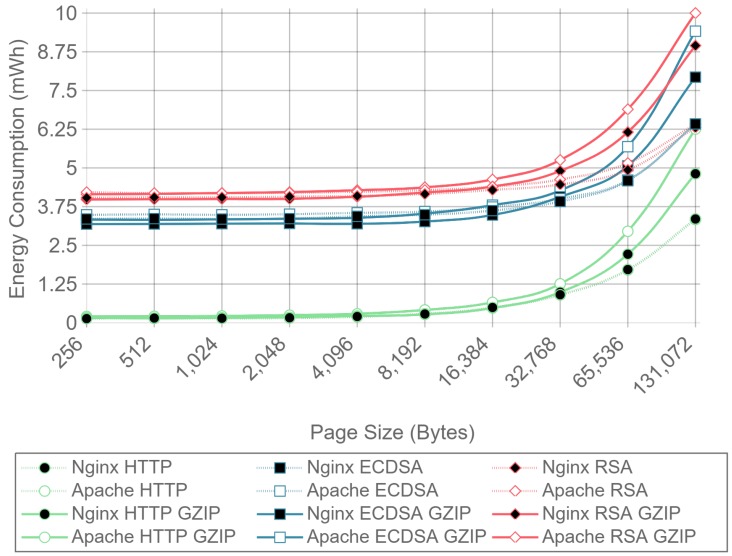
Client-side page size versus energy consumption for Apache2 and Nginx with 16 concurrent clients, with and without GNU ZIP (GZIP) compression.

Similarly, Figure 9 presents the results for the same test, but measuring the energy consumption at the client side. In this case, the difference starts to be noticeable when the payload size exceeds 8192 bytes, although the absolute difference is smaller than in the server case. These differences were expected, since GZIP is asymmetric, the computational cost of compression being greater than the computational cost of decompression. To sum up: GZIP decreases the energy efficiency for large payloads and does not enhance it for smaller payloads.

For a better visualization of the differences in energy consumption, Table 3 and Table 4 present the relative energy consumption, expressed as a percentage, of using GZIP compression compared to no compression. These results correspond to the same test results presented in Figure 8 and Figure 9. For example, in the case of using Apache as a server, Table 3 shows, for a 256-byte payload, that compression makes the system consume 1.9% more in the case of RSA, 4.3% more in the case of ECDSA, and 83.7% more in the case of HTTP. However, when the same tests are run at the client side (whose results are shown in Table 4), it can be observed that the use of compression consumes 1.6% and 4.4% less for RSA and ECDSA, respectively, than when no compression is used. These differences between client and server are explained in Section 4.3 and are due to the asymmetric nature of the compression algorithms. In any case, the gains are minimal and only present at the client side.

With the results presented it can be concluded that, in terms of energy efficiency, GZIP is not recommended for large payloads, and has almost no effect on the payloads that will be typically involved in IoT node communications. For payloads of 128 bytes and less, using GZIP compression will not only increment power consumption, but it will also increase the size of the original data, resulting in more data being transmitted through the network. Because of this, the results presented in the next subsections were obtained without using any kind of compression.

### 4.4. Comparative Analysis of RSA and ECC Energy Consumption and Data Throughput

**Figure 10:**
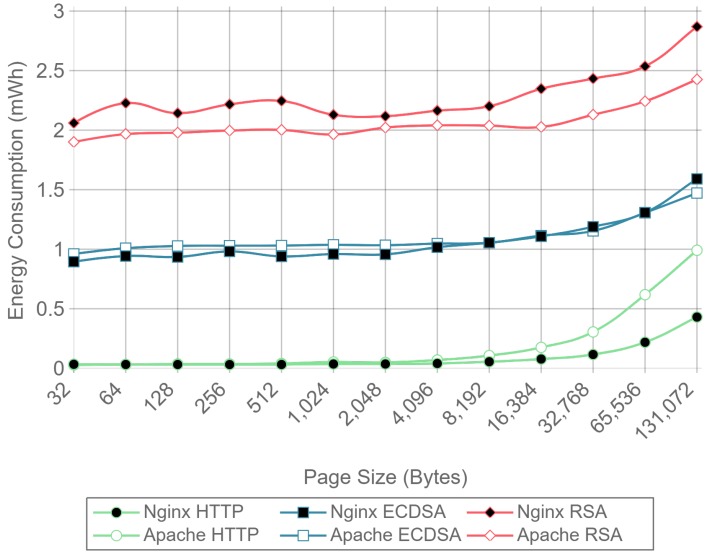
Server-side page size versus energy consumption for Apache2 and Nginx with two concurrent clients.

**Figure 11:**
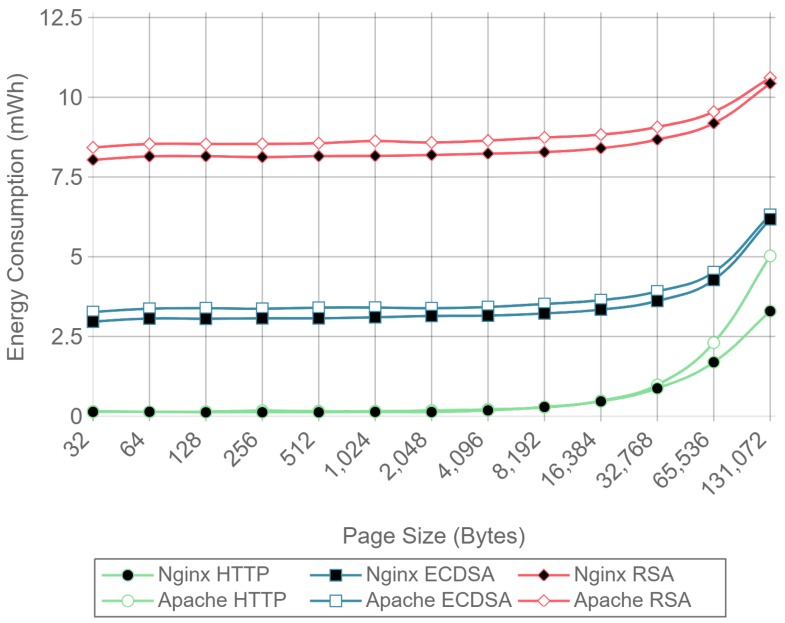
Server-side page size versus energy consumption for Apache2 and Nginx with 16 concurrent clients.

**Figure 12:**
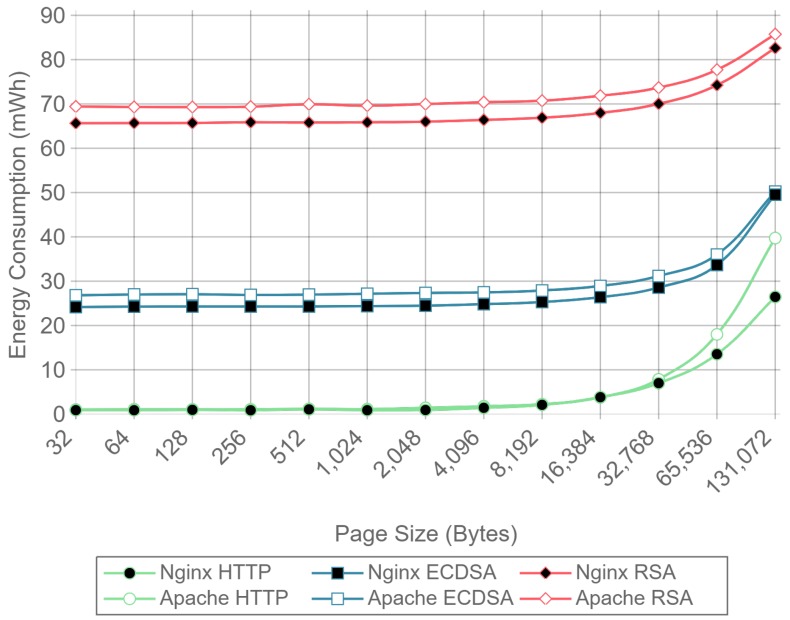
Server-side page size versus energy consumption for Apache2 and Nginx with 128 concurrent clients.

Figure 10, Figure 11 and Figure 12 present the energy consumption on the server-side expressed in mWh for different payloads using 2, 16, and 128 concurrent clients, respectively. These three cases were selected to illustrate low, medium, and high client-transaction load scenarios. Other amounts of concurrent client tests are omitted since their results are very similar. Note that the oscillation of the two-concurrent client curves (Figure 10) is higher than in the other two cases. This is due to the lower number of samples averaged for this case, since only 400 requests are used, in contrast to 3200 and 51,200 requests for the 16 and 128 concurrent client scenarios. Therefore, increasing the number of concurrent clients smooths the results and the differences between the cipher suites due to the larger number of transactions averaged. 

Figure 10 shows that, for two concurrent clients, energy consumption is reduced by half when using ECC. For 16 and 128 concurrent clients, ECC energy consumption savings achieve a 60% reduction respect to the RSA implementation. Note that the gain with two concurrent clients is not larger due to the fact that the SBC processor is more time idle than in the cases when more clients are sending requests. With 16 and 128 concurrent clients, the difference in energy consumption drops to about a 40% reduction for a 128 kilobyte payload. Thus, large payloads increase energy consumption due to internal input–output data operations and network data transmissions. This diminishes the impact of the cipher suite in the energy consumption values, thus reducing the savings and the final difference between cipher suites. Furthermore, by observing the HTTP energy consumption curve of Figure 10, it can be concluded that the effect of large payloads on the energy consumption does not depend on TLS or on the cipher suite selected.

**Figure 13:**
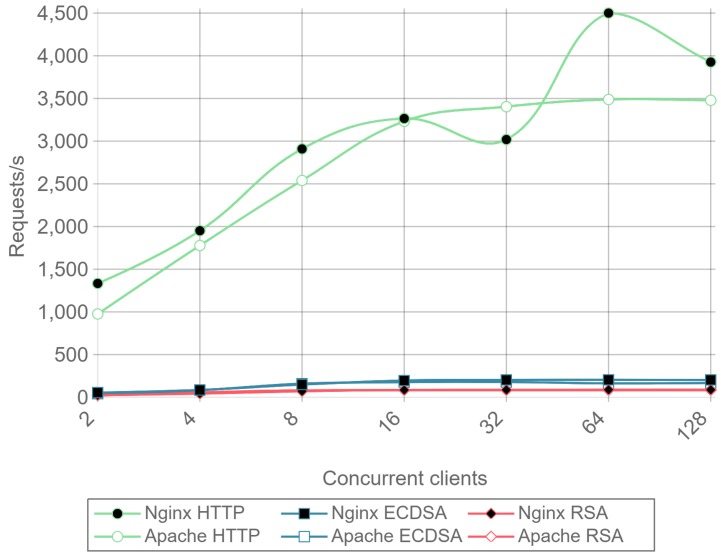
Server-side concurrent clients versus throughput for Apache2 and Nginx with 512 bytes of payload for Elliptic Curve Digital Signature Algorithm (ECDSA), Rivest–Shamir–Adleman (RSA), and HTTP.

**Figure 14:**
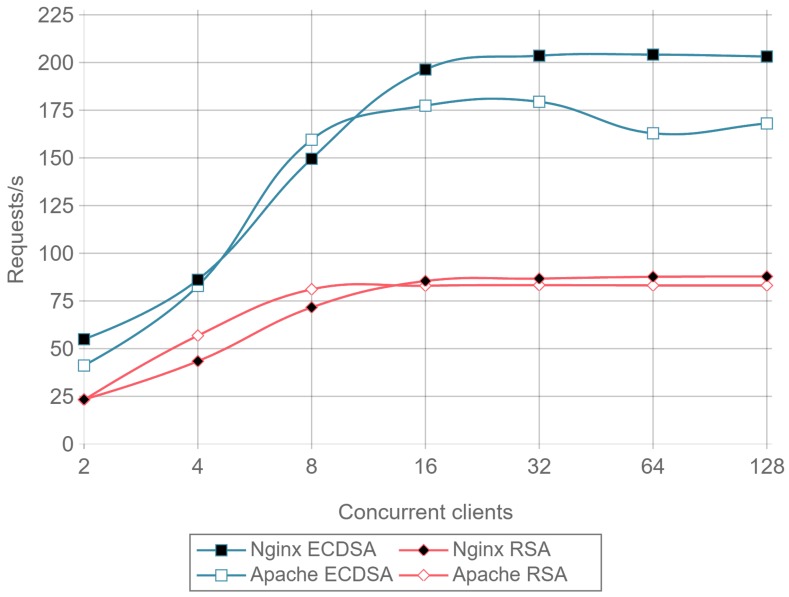
Server-side concurrent clients versus throughput for Apache2 and Nginx with 512 bytes of payload for Elliptic Curve Digital Signature Algorithm (ECDSA) and Rivest–Shamir–Adleman (RSA).

Figure 13 presents the throughput expressed in requests per second for HTTP, ECDSA, and RSA for different amounts of concurrent clients and for a payload size of 512 bytes. Figure 14 presents the same results, but it removes the HTTP curves to observe better the differences between RSA and ECDSA. A drop in performance can be seen for Apache as the number of concurrent clients rises, both for RSA and ECDSA. This figure is a good example of how the implementation and configuration influence the servers and justifies the presence in the comparison of Apache and Nginx instead of showing results for only one server.

Once a minimal number of concurrent clients is reached, in this case 8, the server is constantly responding to requests. This causes the requests per second to remain constant even if the number of concurrent clients is increased. Comparing ECC with RSA, the former is able to handle more than twice requests per second. This can be seen as a relevant result only for servers or IoT gateways that need to handle multiple requests coming from hundreds or even thousands of IoT nodes.

Note that the inverse of requests per second value (i.e., 1/requests/second) represents the mean response time for a single request. When calculating such a metric, a result can be obtained that an RSA transaction takes approximately 0.012 s, while, when using ECC, it only takes 0.005 s. These delay values are critical when real time actuation over IoT devices is required, and, since ECC is 2.5 times faster than RSA, it represents a better option for these types of applications. 

**Figure 15:**
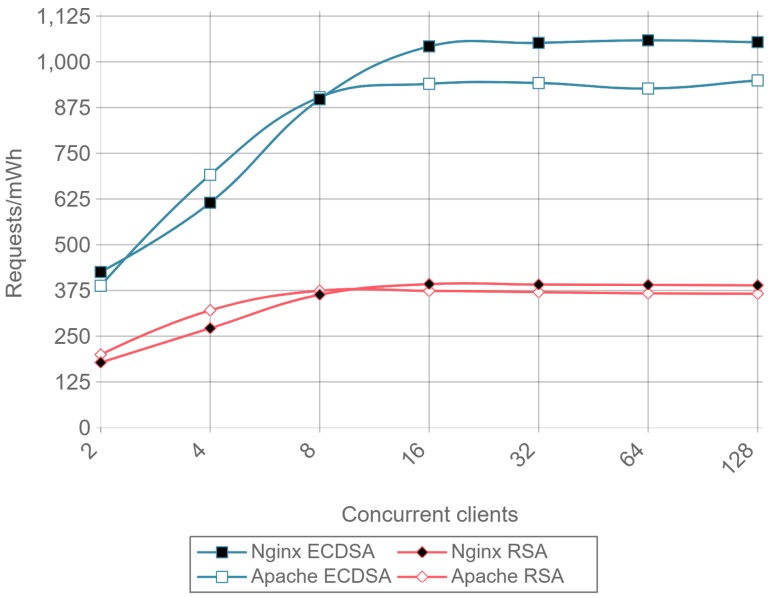
Server-side concurrent clients versus requests/mWh for Apache2 and Nginx with 512 bytes of payload for Elliptic Curve Digital Signature Algorithm (ECDSA) and Rivest–Shamir–Adleman (RSA).

Figure 15 presents the requests per second per mWh (i.e., requests/s/mWh) for a 512-byte payload and different concurrent clients. Once again, it can be observed that ECC consumes less energy than RSA: is requires between 2 and 2.5 times less energy per request than RSA, depending on the total number of concurrent clients. 

**Figure 16:**
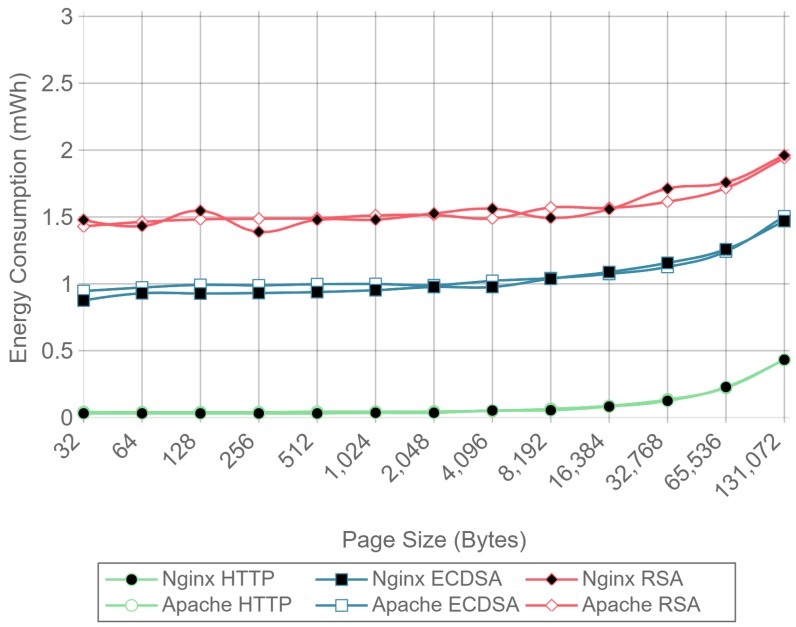
Client-side page size versus energy consumption for Apache2 and Nginx with two concurrent clients.

**Figure 17:**
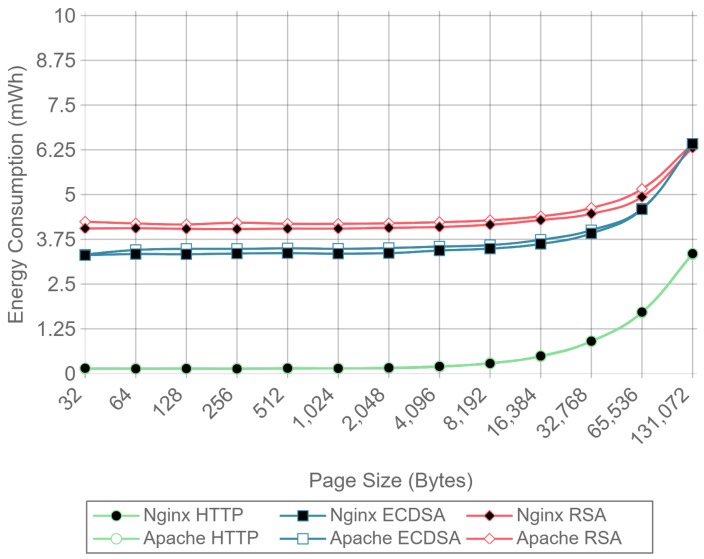
Client-side page size versus energy consumption for Apache2 and Nginx with 16 concurrent clients.

**Figure 18:**
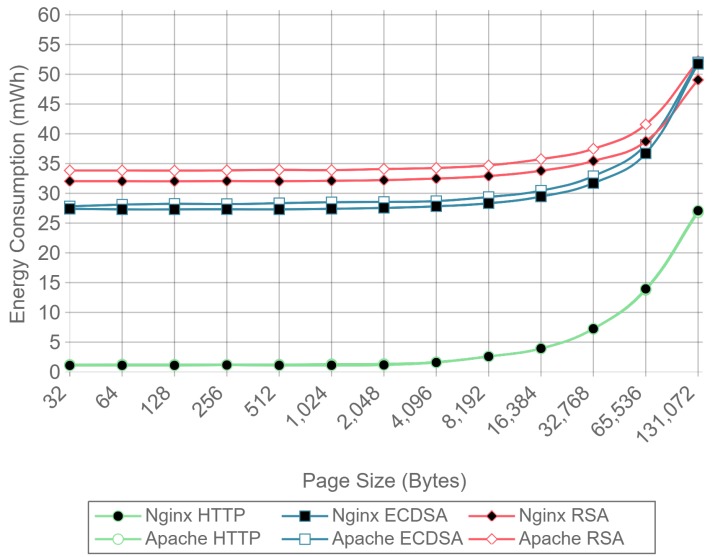
Client-side page size versus energy consumption for Apache2 and Nginx with 128 concurrent clients.

The same power measurements were taken at the client-side. Figure 16, Figure 17 and Figure 18 represent the same results of Figure 10, Figure 11 and Figure 12, but for the client. As it can be observed, at the client-side, ECDSA is still more energy-efficient than RSA, but the difference is less dramatic than at the server-side. This difference is due to the fact that, in the case of RSA, signing and verifying processes require asymmetric computational operations costs, but they are almost symmetric for ECDSA, as other authors have also observed [84]. It is important to note that, in the experiments performed, a 112-bit security level key is being used for RSA in contrast with a more secure 128-bit security level key for ECDSA: even with this difference, ECC is more efficient at both the server- and the client-side. It is also important to remark that, in the tests performed, only server authentication was used. Client authentication might be needed in some scenarios: in such a case, the server- and client-sides would obtain similar energy consumption values, since both carry out signing and verifying operations.

**Figure 19:**
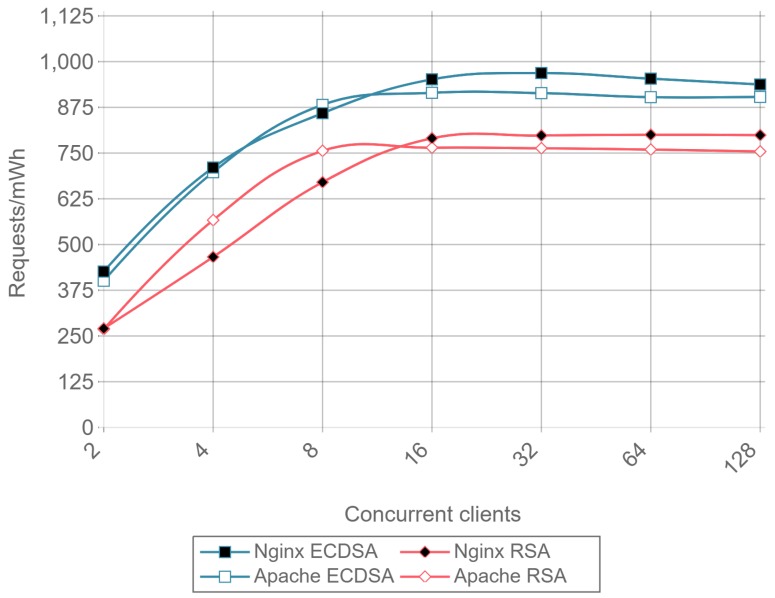
Client-side concurrent clients versus requests/mWh Apache2 and Nginx with 512 bytes of payload for Elliptic Curve Digital Signature Algorithm (ECDSA) and Rivest–Shamir–Adleman (RSA).

Figure 19 shows the results for the same tests as Figure 15, but at the client-side. The results present a reduction in the difference of energy consumption per request between RSA and ECDSA compared to the obtained in server side. However, ECDSA still consumes about 20% less energy per request than RSA.

**Figure 20:**
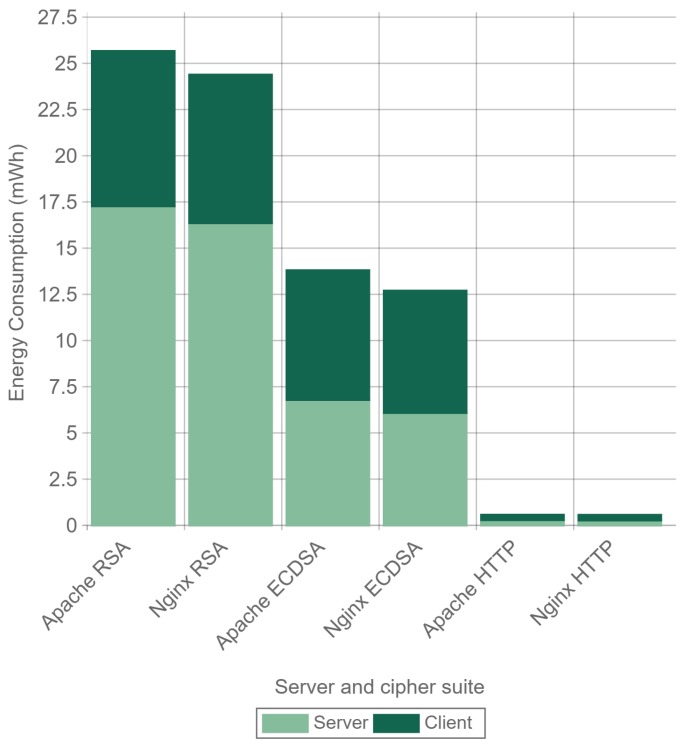
Total energy consumption for a 512-byte payload for 32 concurrent clients. Client and server-side consumption stacked for Apache and Nginx using Elliptic Curve Digital Signature Algorithm (ECDSA), Rivest–Shamir–Adleman (RSA), and plain HTTP.

Finally, Figure 20 illustrates the total energy consumption at server- and client-sides for a 512-byte payload and 32 concurrent users. The total energy consumption is reduced about 50% when using ECDSA instead of RSA. The specific energy consumption values for server- and client-side, expressed in mWh, are shown in Table 5.

### 4.5. Frame Analysis

The main reasons to choose HTTP over other protocols oriented towards energy consumption optimization, like CoAP or Message Queue Telemetry Transport (MQTT), are the repeatability of the tests performed and the need for IoT fog gateways to support TCP/IP. HTTP is the most used protocol on network communications, hence we consider it as the best option in terms of consistency and implementation efficiency. The tests performed were aimed at determining the differences between RSA and ECC when securing network communications. To understand better how the communications protocol and compression affect the tests, as well as how both RSA and ECDSA cipher suites compare to plain HTTP communications, an Ethernet frame analysis was carried out. The IoT scenario remained the same as in the previous tests, but a desktop computer running Wireshark [100] was sniffing the packets exchanged between the Orange Pi PCs. To transmit the frames to the desktop computer, it was necessary to modify the TL-SG108 switch configuration to mirror the packages received in the ports connected to the Orange Pi PCs to the port connected to the desktop computer. Note that the time instants presented in the next figures are not the exact times when the data were received at the Orange Pi PCs, but the instants when the frames reached the desktop computer running Wireshark. While using a dedicated switch and a controlled network environment, these times can vary and have to be treated as approximations.

**Figure 21:**
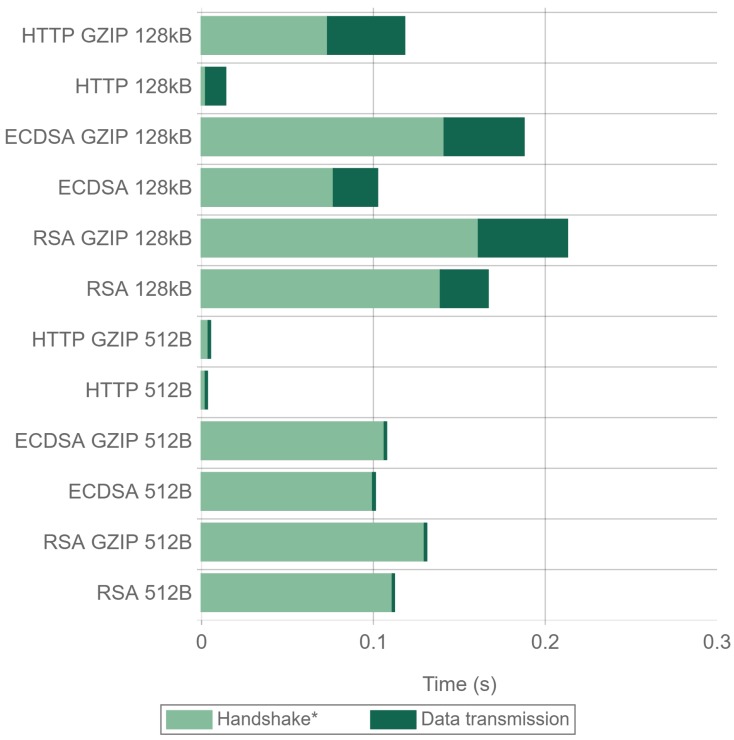
Handshake and payload communication times for 512-byte and a 128-kilobyte payloads using Elliptic Curve Digital Signature Algorithm (ECDSA), Rivest–Shamir–Adleman (RSA), and HTTP, with and without GNU ZIP (GZIP) compression using Apache2. * HTTP handshake for HTTP, Transport Layer Security (TLS) and HTTP handshakes for ECDSA and RSA.

**Figure 22:**
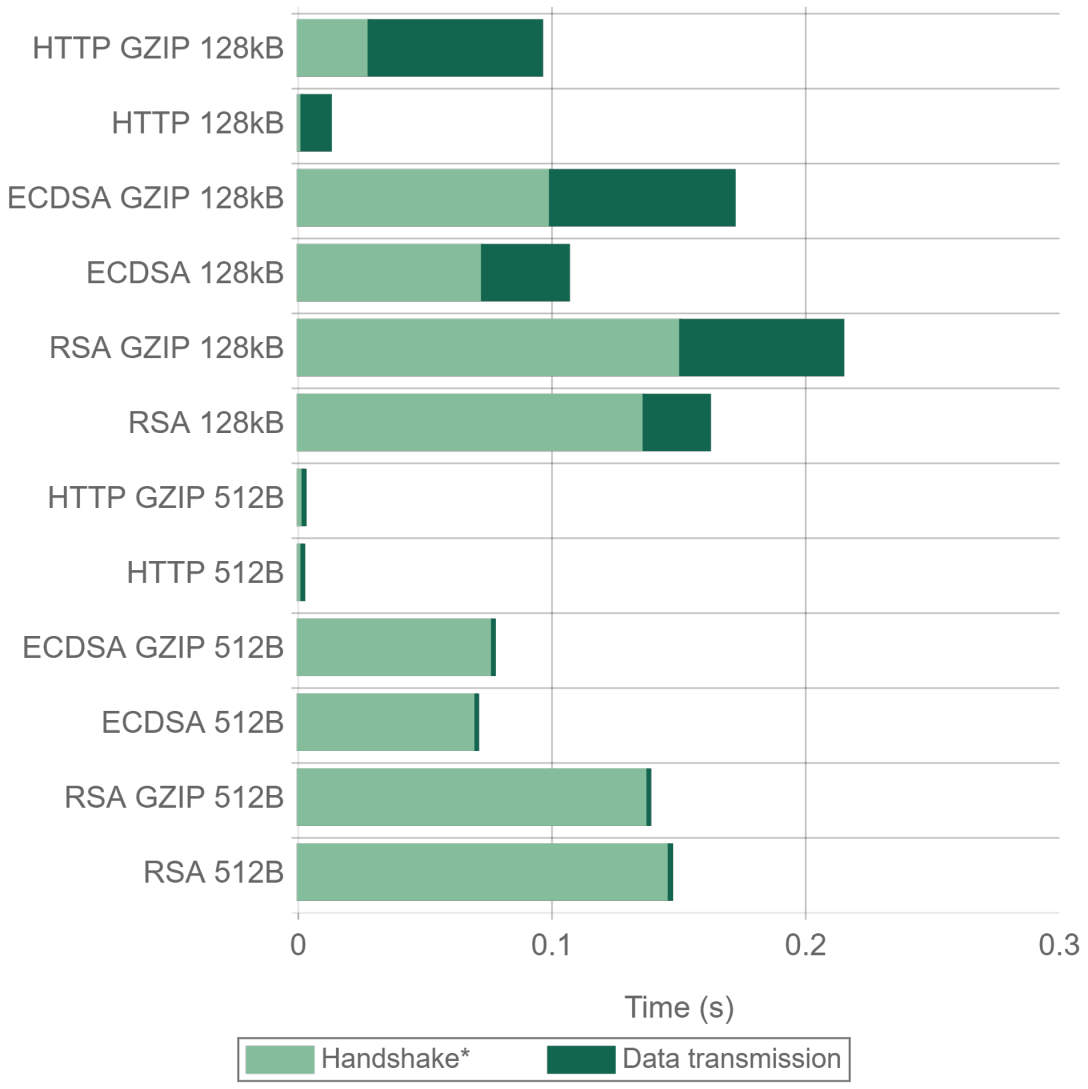
Handshake and payload communication times for 512-byte and a 128-kilobyte payloads using Elliptic Curve Digital Signature Algorithm (ECDSA), Rivest–Shamir–Adleman (RSA), and HTTP, with and without GNU ZIP (GZIP) compression using Nginx. * HTTP handshake for HTTP, Transport Layer Security (TLS) and HTTP handshakes for ECDSA and RSA.

Figure 21 and Figure 22 present the handshake and data transmission times when sending 512 byte and 128 kilobyte packets. The results were obtained using Apache and Nginx for all the cipher suites and compression combinations tested in the previous experiments. In the figures, the handshake time also contains the first compression steps when GZIP is enabled. As it can be seen in the figures, when using GZIP, the first transmitted payload frame takes longer to be sent, since data have to be compressed. The total transmission time is also higher, being more noticeable when using a 128 kilobyte payload. For 512 byte payloads, there is a minimal difference in the total time when using Nginx. Apache presents very similar times, being even faster when RSA is used. For 128 kilobyte payloads, using no compression is always faster, with reductions in time of up to 46%. This makes GZIP not recommended both in terms of energy consumption (as concluded in Section 4.4) and in terms of actuation delay and throughput.

**Figure 23:**
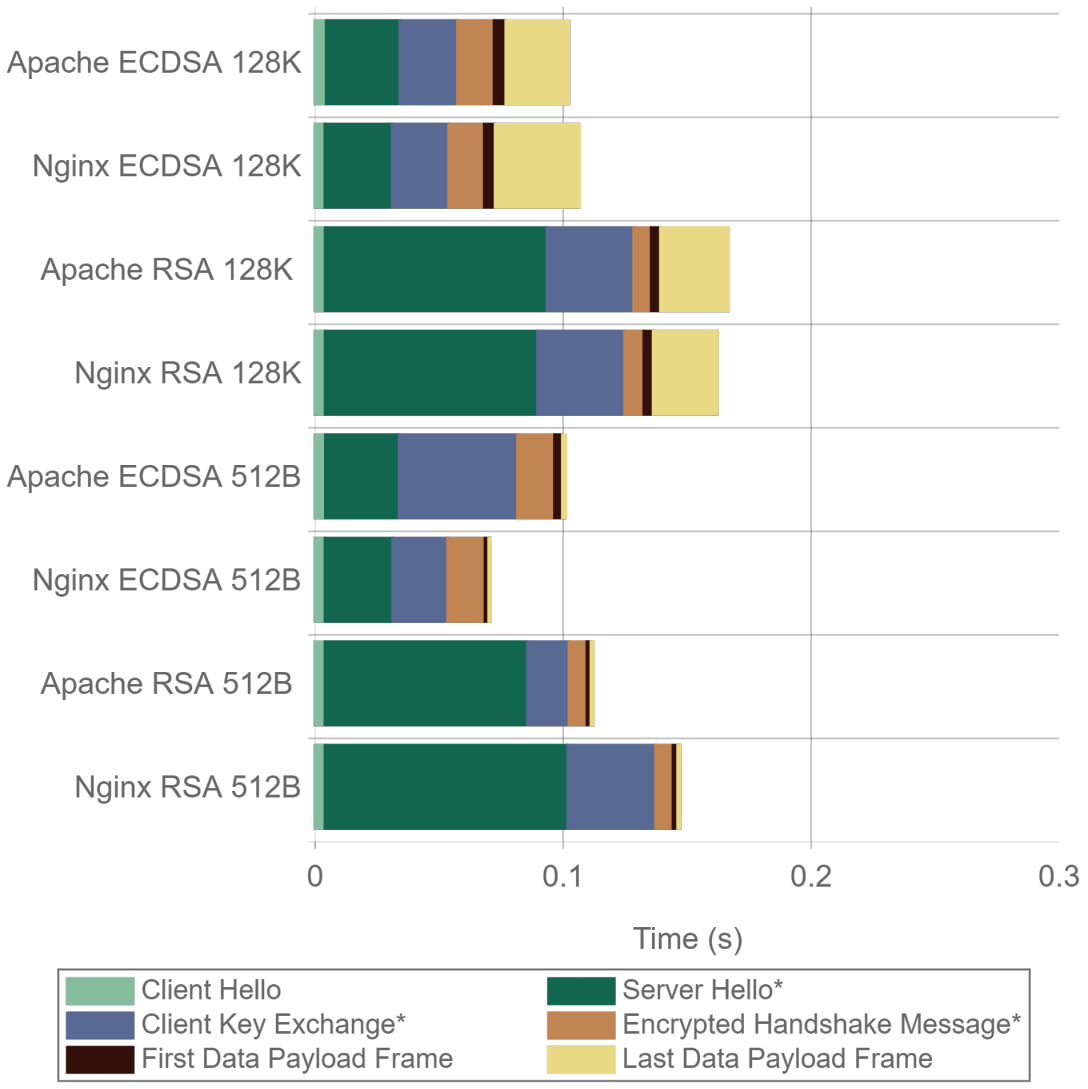
Time elapsed until each main step of the Transport Layer Security (TLS) handshake, start and end of the data transmission. * The frame contains several messages besides the Server Hello/Client Key Exchange/Handshake Messages.

Comparing ECDSA with RSA, it can be seen that, for 128 kilobyte payloads, ECDSA greatly reduces the total transaction time, being the main time reduction produced in the handshake as expected. The differences in the handshake times between GZIP and no GZIP are due to the fact that the time presented for the handshake also includes the first GZIP operations. For 512 byte payloads and using NGINX, the time differences are even larger, since the savings in the handshake remain constant, and the data transmission time is reduced. For Apache and 512 byte payloads, the times are almost the same for all of the tests performed. The reason behind this unexpected behavior seems to be an Apache implementation performance inefficiency in the TLS Handshake when ECDSA is used, as explained in the analysis of Figure 23.

Figure 23 presents a detailed analysis of the time involved in the main TLS handshake steps and data transmission. The size of each colored bar represents the time elapsed from the previous frame. It is important to note that for a 512 byte payload size using Apache with ECDSA, the Client Key-Exchange frame is delayed compared to the other scenarios. This explains why Figure 23 presents very similar time values for ECDSA and RSA, compared with Figure 22, where a clear difference can be seen. It also explains the gap in requests per second between Apache and Nginx that can be seen in Figure 14 and, consequently, the same gap in terms of requests per mWh in Figure 15. The reason for this delay can be attributed to some kind of performance drawback due to Apache implementation or concurrency configuration, although the specific cause remains unknown. Apart from that, it can be observed that, in every scenario, the first frame containing a data payload is always transmitted sooner when using ECDSA.

Table 6 shows the number of total data transmitted through the network, along with the number of frames needed for each server and cipher suite, for a 512 byte and a 128 kilobyte payload, in the cases when GZIP compression is enabled or disabled. Taking a closer look at the no-compression transactions, it can be observed that, for a 512-byte payload, the RSA cipher suite transmits about 15% more data than the ECC cipher suite. Likewise, the ECC cipher suite transmits about 130% more data than plain HTTP.

The number of frames is almost the same in both cipher suites, but such an amount is larger than without security, due to the TLS handshake messages transmitted at the beginning of the communications. However, for a 128 kilobyte payload, the total data transmitted is almost the same for both cipher suites and plain HTTP. Specifically, RSA is the one that requires less frames, followed by ECDSA and HTTP. This behavior is due to cumulative Acknowledgements ACKs, where the same ACK frame is used to confirm several received frames. When using TLS, processing each received frame takes more time than when using plain HTTP. In this interval of time, other frames can arrive, and, in this case, only the ACK is sent to confirm all of them. Thus, the number of ACKs and the total data transmitted are reduced.

As an example, and using the Apache results, if we take the Maximum Transmission Unit (MTU) of our network (1514 bytes of frame length), each TCP frame contains 1448 bytes of payload. For HTTP, only one package of data is sent, corresponding to the 128 kilobyte JSON. This results in approximately 91 TCP segments. Taking into account the handshake (five frames) and FIN message (three frames), a total of 190 frames should be sent if, for each TCP frame, an ACK is also sent, but only 160 frames are transmitted. Using the ECDSA cipher suite, the 128 kilobyte JSON is divided into partitions of 16 kilobytes, corresponding to the TLS Record size, and then each of these partitions is sent over TCP. Since the MTU is 1448 bytes, each partition will need 12 frames to be transmitted (i.e., 16,384/1448=11.31 rounded up to 12 whole frames, since no partial frames can be sent), leaving a total of 96 TCP segments. Once again, taking into account the TLS handshake in this case (10 frames) and the FIN message (six frames), 208 frames would be needed, but only 158 are transmitted. With HTTP, 61 ACKs are sent, and with ECDSA, only 46 are sent, which results in an average of 0.63 ACKs per TCP frame in HTTP and 0.48 ACKs per TCP frame in ECDSA.

Using GZIP compression for a 512 byte payload presents some data reduction and heavily reduces the data and number of frames transferred for a 128 kilobyte payload, but, as it was seen before, these gains in network traffic do not compensate for the cost of compression and decompression in terms of power efficiency.

### 4.6. Comparison to Previous Studies in Terms of Energy Consumption

In this subsection, previous similar studies are analyzed, taking into consideration the results obtained and the testing methodology employed. Main references are presented in Table 7. For each reference, the closest results to our test experiments are presented. Moreover, the table presents a brief description of the used hardware, the algorithms that each solution compares, the energy consumption results and, finally, an evaluation of the methodology employed. In the last column, the resemblance and dissimilarity with our analysis and testing methodology are remarked on in order to perform a fair comparison for each reference.

After analyzing all of them, it can be concluded that none of the references present a fair comparison of ECC and RSA valid for current and future IoT scenarios. The hardware platforms presented were not chosen having an IoT scenario in mind. Some references use outdated algorithms, insecure key sizes, or make unfair comparisons without taking into account security levels. Other authors employ ad hoc implementations that are not useful in real IoT deployments. Various references do not present energy consumption metrics, or the power consumption values are estimated and not measured with precise hardware. Moreover, the vast majority perform isolated tests where no network communications are taken into account.

Contrarily, the tests presented in this paper take into account all of these details. The hardware testbed was chosen to remain comparable with future platforms in the years to come. Furthermore, the cipher suites and the key sizes employed guarantee acceptable security levels for current and future IoT gateway developments. The chosen security library is OpenSSL, an open and widely used implementation of the TLS standard. For instance, the two most extensively used web servers (Apache and Nginx) are employed and compared, and the network communications are carried out over a real network environment. On top of that, energy measurements are performed using isolated and precise hardware with a dedicated SBC.

## 5. Conclusions

In this article, the suitability and possible gains of using ECC digital signing algorithms, instead of the extensively used RSA for securing IoT fog computing gateway communications, was evaluated. It can be stated that even a small difference in energy consumption and computational load results in a huge impact on IoT gateways, especially on the ones with a constrained power supply, so determining the best way of securing their communications is a critical step for a successful and broad deployment.

Therefore, this article made three main contributions aimed at fostering security in resource-constrained IoT gateways. First, in order to establish the basics, it presented a detailed review of the current security, performance capabilities and the problems related to IoT gateways for fog computing. Such a review analyzed the hardware limiting factors, the current state-of-the art hardware platforms and security mechanisms, and its estimated evolution for the near-future, using a general IoT fog computing architecture as a reference. The review was completed with a clear description of TLS. Moreover, the main cipher suites for IoT applications and the state-of-the-art of performance and energy consumption comparisons between ECC and RSA were analyzed. Special care was taken to identify the main limitations when applying the power consumption and performance analysis techniques available in the literature to IoT environments. A detailed explanation of these limitations, and how they are sorted out by the suggested testbed, was also presented.

Second, a hardware testbed was created that allows for using the latest TLS implementations and cipher suites, and performing energy consumption and data throughput tests in a real scenario. The power consumption measurement system guarantees precise and unbiased samples, since it is fully isolated from the rest of the elements being measured. At the same time, the low power consumption and computing capabilities of the Orange Pi PC employed represent the current and short-term future of the energy-efficient IoT gateways that will be essential in low-latency fog computing networks. Moreover, the baseline power consumption test performed proved the suitability of the testbed.

Third, several tests were conducted to evaluate the impact of TLS in IoT communications (between gateways and between the cloud and a gateway), in order to measure the differences between RSA and ECC in terms of security, scalability, power consumption, and data throughput. To make a rigorous comparison in a real-world scenario, the two cipher suites compared (i.e., ECC and RSA) were selected following the NIST guidelines. Nevertheless, the key size used for the ECC cipher suites provides greater level of security than the key size selected for RSA (256-bit ECC provides a strength of 128 while 2048-bit RSA provides a strength of 112).

The experiments made use of two different HTTP servers, with the aim of eliminating any possible bias introduced by the particular server implementation considered. As it is shown in the experiments, there is a small difference in power consumption and throughput between both servers, but a substantial difference can be observed between RSA and ECC across all the tests performed.

ECC greatly outperformed RSA in terms of both power efficiency and communications throughput. In an IoT fog scenario as the one described in Figure 1, the gateways can act both as servers and clients since data requests can be horizontal and vertical, involving IoT nodes or other fog devices. The tests performed allowed for accurately determining the impact of each cipher suite on both sides of the communications. The server-side power consumption when using RSA doubles the one obtained with ECC, while the client-side presents a 15–20% lower power consumption for the ECC scheme. It is important to note that in the tests performed only the server was using a certificate (i.e., only Server Authentication was used), but in most IoT scenarios, client authentication would be also needed. In terms of throughput, ECC is always better than RSA, being able to double the number of requests per second when moderate levels of concurrency are reached.

IoT end devices are usually treated as clients, since the embedded hardware used to implement them is not able to accommodate the necessary certificates and the ciphering algorithms, while maintaining reduced power consumption and reasonable response times. By using ECC, the new emerging hardware platforms for end devices could overcome these limitations and perform as servers or implement client authentication. More work has to be performed to determine the feasibility of using ECC certificates on such devices.

The tests also demonstrated that in the case of transmitting payloads of less than 128 bytes, the use of compression causes larger amounts of data to be transmitted, and, for any of the payload sizes tested, slower transactions and higher power consumption.

A detailed frame analysis showed that the handshake takes more time in the RSA cipher suite than in the ECC one, with remaining data transmission time unaffected. This was the expected result, since both cipher suites only differ in the public key algorithms, the symmetric key and hash algorithms being exactly the same. Therefore, once the TLS Handshake protocol finishes, the TLS Record protocol will perform the same in both cases. Moreover, the total data transmitted is almost the same between both cipher suites, although they differ in the number of frames exchanged due to cumulative ACKs.

After all the experiments were performed, it can be concluded that, in specific resource-constrained IoT scenarios where energy efficiency and throughput are essential, ECC cipher suites should replace RSA cipher suites. Moreover, for the security levels required nowadays, measurements indicate that ECC obtains power consumption reductions of up to 50% and a data throughput that doubles RSA in most scenarios. Furthermore, considering the near-term prospects of more secure levels, the key sizes needed in RSA will make it impractical not only for IoT fog computing applications, but for any secure connection.

## Figures and Tables

**Table 1:** Characteristics of some of the latest Internet of Things (IoT) development boards.

Name	Clock Rate	Cores	RAM	References
Meshlium 4.0	1 GHz	4	2 GB	[32]
UDOO X86 Basic	2.00 GHz	4	2 GB	[33]
Raspberry Pi3	1.2 GHz	4	1 GB	[34,35]
Tessel 2	580 MHz/48 MHz	1/1	64 MB	[36]
UDOO NEO BASIC	1 GHz/200 MHz	1/1	512 MB	[37]
BeagleBoneBlack	800 MHz	1	512 MB	[38]
Intel Edison Module	500 MHz	2	1 GB	[39]
Arduino Yún	400 MHz/16 MHz	1/1	64 MB/2.5 KB	[40,41]
Arduino TIAN	560 MHz/48 MHz	1/1	64 MB/32KB	[42]
ESP32	240 MHz	2	512 KB	[43]
Particle Photon	120 MHz	1	128 KB	[44]

**Table 2:** Comparable strengths for symmetric, Rivest–Shamir–Adleman (RSA) and Elliptic Curve Cryptography (ECC) ciphers.

Security Level	Symmetric Key Algorithms	RSA Key Size	ECC Key Size
80	2TDEA	1024 bits	160–223 bits
112	3TDEA	2048 bits	224–255 bits
128	AES-128	3072 bits	256–383 bits
192	AES-192	7680 bits	384–511 bits
256	AES-256	15,360 bits	512+ bits

**Table 3:** Server-side page size versus energy consumption difference (in percentage) between GNU ZIP (GZIP) compression and no compression for Apache2 and Nginx with 16 concurrent clients.

Bytes	Apache RSA	Nginx RSA	Apache ECDSA	Nginx ECDSA	Apache HTTP	Nginx HTTP
256	1.9%	1.7%	4.3%	1.1%	83.7%	39.8%
512	1.7%	0.9%	3.5%	1.5%	112.2%	34.3%
1024	1.1%	1.6%	4.2%	0.6%	136.4%	25.0%
2048	2.8%	2.3%	5.5%	0.4%	131.2%	68.2%
4096	2.8%	2.5%	8.0%	1.8%	140.1%	69.1%
8192	5.0%	4.6%	11.5%	3.8%	187.0%	72.4%
16,384	10.0%	8.9%	22.6%	13.2%	179.7%	117.0%
32,768	23.4%	18.9%	54.9%	38.2%	197.9%	179.5%
65,536	58.0%	55.7%	125.7%	93.3%	209.3%	291.1%
131,072	118.2%	122.3%	198.0%	146.2%	209.8%	337.4%

**Table 4:** Client-side page size versus energy consumption difference (in percentage) between GNU ZIP (GZIP) compression and no compression for Apache2 and Nginx with 16 concurrent clients.

Bytes	Apache RSA	Nginx RSA	Apache ECDSA	Nginx ECDSA	Apache HTTP	Nginx HTTP
256	−1.6%	−1.3%	−4.4%	−5.0%	46.0%	19.2%
512	0.5%	−1.5%	−5.0%	−5.2%	48.6%	−3.2%
1024	0.1%	− 1.3%	−4.3%	−4.5%	50.6%	11.4%
2048	0.5%	−1.7%	−4.2%	−4.7%	60.8%	10.4%
4096	1.2%	−0.6%	−4.5%	−7.1%	50.6%	9.8%
8192	2.1%	1.1%	−1.8%	−6.3%	41.3%	−4.4 %
16,384	5.3%	2.6%	1.6%	−3.9%	37.0%	−3.4%
32,768	13.6%	9.8%	6.7%	3.8%	40.1%	8.8%
65,536	33.7%	24.8%	23.4%	10.5%	73.2%	28.9%
131,072	61.3%	41.9%	46.7%	23.5%	87.8%	43.6%

**Table 5:** Total energy consumption for a 512-byte payload for 32 concurrent clients. Client and server-side consumption for Apache and Nginx using Elliptic Curve Digital Signature Algorithm (ECDSA), Rivest–Shamir–Adleman (RSA), and plain HTTP.

Server and Cipher ${}^{1}$	Server Consumption (mWh)	Client Consumption (mWh)
Apache RSA	17.28	8.38
Nginx RSA	16.36	8.02
Apache ECDSA	6.79	7.00
Nginx ECDSA	6.08	6.60
Apache HTTP	0.28	0.27
Nginx HTTP	0.26	0.28

${}^{1}$ Cipher or plain HTTP.

**Table 6:** Total data and frames transmitted using GNU ZIP (GZIP) and no compression for 512 byte and 128 kilobyte payloads.

	No Compression				GZIP			
	512 Bytes		128 Kilobytes		512 Bytes		128 Kilobytes	
Server and Cipher ${}^{1}$	Bytes	Frames	Bytes	Frames	Bytes	Frames	Bytes	Frames
Apache RSA	4206	16	142,303	127	4141	16	85,777	90
Nginx RSA	4104	15	142,428	129	3957	15	85,177	91
Apache ECDSA	3626	16	143,770	158	3561	16	85,395	93
Nginx ECDSA	3524	15	143,366	152	3438	16	84,598	91
Apache HTTP	1613	10	142,081	160	1519	10	83,272	95
Nginx HTTP	1531	9	144,263	163	1450	9	83,346	98

${}^{1}$ Cipher or plain HTTP.

**Table 7:** Analysis of the main RSA and ECC comparisons available.

Ref.	Hardware	Algorithms Characteristics	Main Results	Methodology Evaluation
[84]	Compaq iPAQ H3670 SA-11000 @ 206 MHz/64 MB RAM	RSA-RC5-SHA1 and ECC-AES-MD5	Energy consumption savings of 16.7 % for a 7.9KB payload when using the ECC cipher suite compared to the RSA one.	Outdated cipher suites, key sizes are not explicitly specified.
[24]	Mica2dot sensor platform, ATmega128L @ 4 MHz	Handshake protocol based on 1024-bit RSA and 160-bit ECC	Energy consumption savings of 76.2 % when using ECC-160 compared to RSA-1024.	Insecure key sizes. Ad-hoc and simplified key-exchange algorithm and certificates.
[82]	BeagleBoard, Dual Core ARM Cortex-M3 @ 200 MHz/ 512 MB RAM	192, 224 and 521-bit ECC and 192, 256 and 512-bit RSA	Up to 93.02 % reduction of energy consumption when using ECC compared to RSA.	It is a direct comparison between both algorithms using the same key sizes. The relative security levels are not taken into account. Energy consumptions are aggregated for each algorithm, without presenting individual results for the different key sizes employed. Only key generation is measured.
[101]	STRONGARM CPU @ 206 MHZ	2240-bit RSA and 233-bit ECDSA	ECDSA key generation found to be more than 73 times faster than RSA. Total time of generation and verification found to be almost 16 times faster for ECDSA compared to RSA.	It is focused on time measurements, no power consumption metrics presented.
[102]	Personal Computer with Core 2 Duo CPU @ 2.0 GHz/4 GB RAM	2048-bit RSA and 163-bit ECDSA	Up to 4 times faster TLS-OBC [103] certificate generation when using ECDSA compared to RSA.	Only client authentication times are measured. Personal computer environment. No energy consumption metrics presented.
[104]	Intel XScale PXA250 @ 400 MHz/64 MB RAM and Intel XScale PXA270 @ 624 MHz/64 MB RAM	2048-bit RSA and 224-bit ECDSA	Up to 78 % energy consumption reduction when using ECDSA compared to RSA for PXA250. Only 18 % energy consumption savings for PXA270.	This solution tests cryptographic algorithms but no actual network transactions involving them are conducted. Power consumption is estimated with reference values obtained from battery status information using a software approach without external hardware. Battery status information is obtained using the same device that runs the cryptographic algorithm.

## Abbreviations

ACPI	Advanced Configuration and Power Interface
AES	Advanced Encryption Standard
CA	Certification Authority
DH	Diffie–Hellman
DHE	Ephemeral Diffie–Hellman
DSA	Digital Signature Algorithm
DTLS	Datagram Transport Layer Security
ECC	Elliptic Curve Cryptography
ECDHE	Elliptic Curve Diffie–Hellman Ephemeral
ECDSA	Elliptic Curve Digital Signature Algorithm
FTPS	File Transfer Protocol over SSL
GCM	Galois/Counter Mode
HMAC	Hash Message Authentication Code
IMAPS	Internet Message Access Protocol over SSL
IoT	Internet of Things
KDF	Key Derivation Function
MAC	Message Authentication Code
MitM	Man-in-the-Middle Attack
NIST	National Institute of Standards and Technology
PFS	Perfect Forward Secrecy
PUF	Physical Unclonable Functions
RSA	Rivest–Shamir–Adleman
RTSP	Real Time Streaming Protocol
SBC	Single Board Computer
SHA	Secure Hash Algorithm
SMTPS	Simple Mail Transfer Protocol Secure
SSL	Secure Socket Layer
TDES	Triple Data Encryption Standard
TDEA	Triple Data Encryption Algorithm
TLS	Transport Layer Security
WSN	Wireless Sensor Network
